# Near full genome characterization of HIV‐1 unique recombinant forms in Cameroon reveals dominant CRF02_AG and F2 recombination patterns

**DOI:** 10.1002/jia2.25362

**Published:** 2019-07-28

**Authors:** Andrew N Banin, Michael Tuen, Jude S Bimela, Marcel Tongo, Paul Zappile, Alireza Khodadadi‐Jamayran, Aubin J Nanfack, Iheanyi O Okonko, Josephine Meli, Xiaohong Wang, Dora Mbanya, Jeanne Ngogang, Miroslaw K Gorny, Adriana Heguy, Charles Fokunang, Ralf Duerr

**Affiliations:** ^1^ Department of Pathology New York University School of Medicine New York NY USA; ^2^ Faculty of Medicine and Biomedical Sciences University of Yaoundé 1 Yaoundé Cameroon; ^3^ Faculty of Science Department of Biochemistry University of Yaoundé 1 Yaoundé Cameroon; ^4^ Center of Research for Emerging and Re‐Emerging Diseases (CREMER) Institute of Medical Research and Study of Medicinal Plants Yaoundé Cameroon; ^5^ Applied Bioinformatics Laboratories (ABL) and Genome Technology Center (GTC) Division of Advanced Research Technologies (DART) New York University Langone Medical Center New York NY USA; ^6^ Medical Diagnostic Center Yaoundé Cameroon; ^7^ Chantal Biya International Reference Center for Research on HIV/AIDS Prevention and Management Yaoundé Cameroon; ^8^ Virus Research Unit Department of Microbiology University of Port Harcourt Port Harcourt Nigeria; ^9^ Manhattan Veterans Affairs Harbor Healthcare Systems New York NY USA

**Keywords:** unique recombinant forms, near full genome sequencing, third‐generation sequencing, intra‐patient viral diversity, Env epitopes and drug resistance mutations

## Abstract

**Introduction:**

In Cameroon, a manifold diversity of HIV strains exists with CRF02_AG and unique recombinant forms (URFs) being the predominant strains. In recent years, a steady increase in URFs and clade F2 viruses has been monitored through partial genome sequencing. There is an information gap in the characterization of emerging URFs along the full genome, which is needed to address the challenges URFs pose towards diagnosis, treatment and HIV‐1 vaccine design.

**Method:**

Eighteen Cameroonian URFs from samples collected between the years 2000 and 2015 were studied using a newly developed near full genome sequencing (NFGS) protocol based on variable nested RT‐PCRs with a versatile primer set. Near full genomes were characterized for recombination patterns and sequence signatures with possible impact on antiretroviral treatment or Env‐directed immune responses. Third‐generation sequencing (3GS) of near full or half genomes (HGs) gave insight into intra‐patient URF diversity.

**Results:**

The characterized URFs were composed of a broad variety of subtypes and recombinants including A, F, G, CRF01_AE, CRF02_AG and CRF22_01A1. Phylogenetic analysis unveiled dominant CRF02_AG and F2 recombination patterns. 3GS indicated a high intra‐patient URF diversity with up to four distinct viral sub‐populations present in plasma at the same time. URF *pol* genomic analysis revealed a number of accessory drug resistance mutations (DRMs) in the ART‐naïve participants. Genotypic *env* analysis suggests CCR5 usage in 14/18 samples and identified deviations at residues, critical for gp120/gp41 interphase and CD4 binding site broadly neutralizing antibodies in more than half of the studied URFs. V1V2 sites of immune pressure in the human RV144 vaccine study varied in more than a third of URFs.

**Conclusions:**

This study identified novel mosaic patterns in URFs in Cameroon. In line with the regional predominance of CRF_02AG and the increased prevalence of clade F2, prominent CRF_02AG and F2 background patterns were observed underlying the URFs. In the context of the novel mosaic genomes, the impact of the identified accessory DRMs and Env epitope variations on treatment and immune control remains elusive. The evolving diversity of HIV‐1 URFs in Cameroon requires continuous monitoring to respond to the increasing challenges for diagnosis, antiretroviral treatment and prevention.

**Abbreviation**
3GS: third‐generation sequencing
cDNA: complementary deoxyribonucleic acid
CRF: circulating recombinant form
GTR: generalized time reversible
HG: half genome
MDC: medical diagnostic center
NFG: near full genome
NFGS: near full genome sequencing
NYUSoM: New York University School of Medicine
PCR: polymerase chain reaction
RNA: ribonucleic acid
RT: reverse transcriptase
SGA: single genome amplification
URF: unique recombinant form

## 1 Introduction

Based on the latest HIV molecular surveillance report, recombinant forms account for almost every fourth HIV infection globally (22.8%) 1. Circulating recombinant form (CRF) 02_AG (CRF02_AG) is the most predominant recombinant form and the fourth most predominant lineage in the world (7.7%). Also, there has been a consistent and ongoing rise in the percentage of unique recombinant forms (URFs) globally, which currently constitute the fifth most prevalent group of HIV viruses (6.1%) 1. Comparing the periods 1990 to 1999 and 2010 to 2015, URFs almost doubled (1.7x) globally and increased more than four‐fold (4.6x) in West Africa 1. The Congo basin, located in West‐Central Africa between the Sanaga River in Cameroon and the Congo River in the former Belgian Congo, is hypothesized to be the geographical origin of HIV‐1. Cross‐species transmission events from chimpanzees and other monkeys to hunters and butchers are supposed to be initiators of the HIV epidemic in humans 2, 3, 4, 5. Cameroon is the likely country of origin of HIV‐1 groups M, N, O and P 6, 7, 8. Possibly as a consequence, it has one of the most genetically diverse HIV epidemics in the world 9, 10, 11. The predominant lineage is the recombinant CRF02_AG, which accounts for more than half of infections 12, 13. Also, circulating virus lineages include virtually every known HIV‐1 group M (HIV‐1 M) pure subtype, many CRFs, and a variety of URFs composed of pure, CRF and/or non‐classifiable sequences making this country a unique source of rare and emerging HIV‐1 M viral variants 5, 9, 10, 11, 12, 13, 14, 15, 16, 17, 18. The range of subtypes and the extent of viral diversity within a geographical region considerably impact HIV diagnosis, treatment and prevention 19. It is, therefore, crucial to monitor and genetically characterize HIV globally, specifically at HIV diversity hot spot regions like Cameroon.

The characterization of circulating HIV‐1 M strains in Africa is incomplete, particularly in West‐Central Africa, where the diversity of subtypes is exceptionally high. A crucial genetic feature of HIV is its recombination‐prone nature leading to the emergence of intra‐ and inter‐subtype recombinants in dually infected individuals 20, 21. Currently, there are at least 98 CRFs 22, and numerous URFs identified, composed of pure and other recombinant subtypes including under‐sampled parental lineages that cannot be reasonably classified within the established HIV‐1 M subtypes 23, 24, 25. Recombination‐prone sites have been identified 26, 27; however, recombination events are scattered along the whole HIV genome 28. Most genotypic HIV‐1 M data is determined through partial genomic sequencing of one or a few limited regions. Partial genome sequencing bears the risk of missing recombination sites and parental subtypes, which results in an inaccurate or obscured determination of HIV‐1 M genetic diversity.

As part of our ongoing molecular surveillance efforts in Cameroon, our laboratories have in recent years identified and characterized two CRFs (CRF_36cpx and CRF_37cpx) 29, 30, and we have monitored a steady increase in URFs 12, 13, 17, 21. Through the application of a robust and versatile near full genome (NFG) amplification and sequencing technique 31, the current study characterized 18 near‐full genomes of HIV‐1 M recombinants. Deep sequencing revealed considerable intra‐patient URF diversity and evolution. Near full genome sequencing (NFGS) enabled the mapping of sites targeted by antiretroviral therapy or neutralizing antibodies, for example, the *pol* and *env* genomic regions, for resistance‐conferring mutations. The study highlights the broad utility of URF NFGS for HIV surveillance, diagnosis and personalized therapy.

## 2 Methods

### 2.1 Ethical clearance

This study was approved by the Institutional Review Boards of the Cameroon Ministry of Public Health and the New York University School of Medicine (NYUSoM), New York, USA. Before sample collection, informed consent was obtained from the study participants, who were all part of a cohort of HIV positive individuals; this cohort is monitored at the Medical Diagnostic Center (MDC), Yaoundé, Cameroon in collaboration with NYUSoM, New York, USA.

### 2.2 Study samples

Whole blood samples were collected between the years 2000 and 2015. They were shipped from the MDC under regulatory guidelines of transfer on biological samples to NYUSoM, where plasma was separated from peripheral blood mononuclear cells through Ficoll gradient centrifugation (Histopaque, Sigma‐Aldrich, St. Louis, MO, USA), and stored at −80°C. Eighteen URFs were selected based on previously published work from our group using partial genome sequencing (total of 509 participants) 11, 12, 13, 17, 32, 33. Inclusion criteria were genetic evidence of URF infection and available plasma volumes ≥500 μL. RNA extraction, cDNA synthesis and NFGS using Sanger sequencing or third‐generation sequencing (3GS) were performed as described in detail in Banin *et al*. 31. Briefly, a flexible one, two, or multiple amplicon strategy enabled NFGS for all study samples. Amplifications and sequencing were performed using a newly composed set of primers binding to semi‐conserved regions within the HIV‐1 genome of group M viruses. Single genome amplifications (SGA) on endpoint‐diluted cDNA were performed for six near full or half genomes (HGs) to preclude that the identified URFs were the result of amplification artefacts based on template switching when mixtures of genetically diverse templates were present 31.

### 2.3 Phylogenetic analysis

Generated sequences were assembled using DNA Star SeqMan Pro (where applicable) and aligned using CLUSTAL Omega (https://www.ebi.ac.uk/Tools/msa/clustalo/) with reference sequences from all known HIV‐1 M (sub‐)subtypes and CRFs from the Los Alamos National Library (LANL) HIV sequence database 34. Maximum likelihood phylogenetic trees were generated using MEGA version 5.2 software package with 1000 bootstrap replicates, and pairwise evolutionary distances were estimated using the maximum composite likelihood substitution model 35. Intra‐patient diversity was studied based on 3GS data as follows. After removal of short and intermediate 3GS reads (Table 5 in Banin *et al*. 31), the long reads were aligned in MEGA5.2 and phylogenetically analysed for cluster formation. For confirmation, multiple reads per cluster were analysed using highlighter plots and Simplot for consistent breakpoint patterns within each cluster and differing breakpoint patterns between clusters. 3GS (sub)populations were averaged to consensus (con) sequences using Consensus Maker 34 or SeqMan Pro.

### 2.4 Highlighter

The highlighter tool of the LANL database 34 was used to analyse the site‐specific genetic diversity between subjects and between different reads per subject along the entire study sequence. It was also used to detect/exclude cross‐contamination between samples.

### 2.5 Recombination analysis

Each query sequence was plotted against all known pure HIV‐1 M subtypes and CRF reference sequences in Similarity plots (Simplot software package 3.5.2, window size 200, step size 20). Both similarity plots and the recombinant identification program (RIP; LANL database) were used to identify the contributing parental subtypes of the recombinants (Figure S1). A more focused recombination breakpoint analysis was done by bootscanning (Simplot, window size 200, step size 20, 250 bootstrap replicates). Bootstrap support of 70% between a subtype reference strain and a query sequence was used as criteria to assign a subtype to a breakpoint region. If bootstrap values remained below 70% for all pure and CRF reference subtypes, the region was considered unidentified.

Recombinant fragments were phylogenetically studied using RAxML version 8 36 implemented in CIPRES 37. An un‐rooted maximum likelihood phylogenetic tree was constructed from an alignment (created with MUSCLE, implemented in MEGA version 5.2) combining URF recombinant fragments of sizes >900 bp and full genome reference sequences with 1000 bootstrap replicates. RAxML is based on generalized time reversible (GTR) nucleotide substitution models (GTR‐GAMMA used in this study) and enables accurate inference of phylogenies from alignments containing sequences of different sizes, i.e. it tolerates large amounts of missing data 36, 38. It was suited to perform phylogenetic subtype determination/confirmation for several recombinant fragments >900 bp in size (as determined using similarity plots and bootscanning) in a single maximum likelihood phylogenetic tree.

### 2.6 Drug resistance mutation and co‐receptor tropism analysis

NFGS were tested for canonical HIV‐1 drug resistance mutations (DRM) using the Stanford HIV drug resistance database (https://hivdb.stanford.edu/) with consideration of recent literature and updates of the World Health Organization (WHO) and the International Antiviral Society (IAS)‐USA 39, 40, 41, 42. The analysis included the *pol* genomic regions encoding for protease (PR), reverse transcriptase (RT) and integrase (IN). Co‐receptor tropism analysis was performed using the Geno2pheno co‐receptor 2.5 tool (http://coreceptor.geno2pheno.org/) with a 5% false positive rate, as recommended for the prediction of CRF02_AG strains 43, and the PhenoSeq tool for (sub‐)subtypes A/A1/A2/CRF02_AG (http://tools.burnet.edu.au/phenoseq/) 44.

### 2.7 Env epitope and N‐glycosylation analysis

Env amino acid sequences were screened for the presence of key residues for broadly neutralizing antibodies and for sites of immune pressure identified in the partially protective human vaccine study RV144 45, 46. Env amino acid sequences from the study subjects and reference sequences of subtype B (HXB2), CRF02_AG (0014BBY) and F2 (CM53657) were aligned using Clustal Omega. Potential N‐linked glycosylation sites were determined using the N‐Glycosite tool from the LANL database 34 with sites highlighted in red (Figure S8). Key N‐glycosylation sites, residues that are critical for broadly neutralizing antibodies and sites of immune pressure based on the RV144 vaccine study, were labelled according to Rolland *et al*. 46, deCamp *et al*. 47 and Courtney *et al*. 21.

### 2.8 Data storage and documentation

NFG sequences are available from GenBank with accession numbers MK086109‐MK086126. Whole sets of raw deep sequencing reads have been deposited in the Sequence Read Archive with accession numbers SRR9074509‐SRR9074519. All other data are available from the corresponding author upon reasonable request.

## 3 Results

### 3.1 URFs in Cameroon are composed of diverse subtypes with scattered recombination breakpoints

Plasma samples from 18 Cameroonian study participants at ART‐naïve time points were selected for the current study (Table 1). All study participants were infected with HIV‐1 M URFs based on partial genome sequencing 11, 12, 17, 32, 33; no further biological selection criteria were applied. The study included participants from both genders, between 20 and 70 years of age, and at different stages of HIV‐1 infection. The samples were collected between the years 2000 and 2015 with plasma viral loads between 300 and 950,000 copies/mL. CD4 counts were in the range between 170 and 530 cells/μL. The clinical and demographic data of the participants and a summary of the NFGS approaches are shown in Table 1.

**Table 1:** Clinical and demographic data of study participants and experimental NFGS data

#	Sample ID	Sex	Age	Sampling date	Diagnosis date	CD4 counts (cells/μL)	Viral load copies/mL	Antiretroviral treatment	# Amplicons for NFGS	Genotype (NFGS)
1	LB016‐1	F	39	January 2010	November 2007	201	9870	Naïve	1	02AG/A1
2	LB069‐1	F	36	May 2010	July 2008	266	25,618	Naïve	1	22_01A1/F2
3	LB082‐1	M	43	June 2010	2006	226	953,760	Naïve	2	A1/F2
4	LB089‐1	M	30	July 2010	June 2010	371	20,148	Naïve	1	02AG/F2
5	LB095‐1	M	21	July 2010	July 2010	443	78,218	Naïve	1	02AG/01AE
6	LB104‐1	F	41	October 2010	October 2010	407	47,366	Naïve	2	02AG/F2/22_01A1
7	MDC 131‐1	F	48	June 2011	NA	529	4140	Naïve	2	02AG/F2
8	MDC 179‐2	M	39	June 2012	July 2011	306	3620	Naïve	2	02AG/F2
9	BDHS024‐2	F	27	NA	NA	NA	4198	Naïve	2	02AG/F2
10	BDHS33	F	20	NA	November 2006	NA	28,480	NA	3	02AG/F2
11	NYU119‐3	NA	NA	NA	NA	NA	NA	NA	2	02AG/F2
12	NYU124‐2	F	35	September 2001	January 2001	495	5688	Naïve	2	02AG/F2
13	NYU129‐5	F	44	August 2002	January 2000	426	478,774	Naive	1	02AG/A1
14	NYU1122‐1	M	70	Jun 2000	June 2000	NA	306	Naïve	1	02AG/A1
15	NYU1999‐1	F	23	July 2000	July 2000	NA	14,558	Naïve	2	02AG/F2
16	NYU2140‐1	F	50	July 2000	July 2000	NA	13,564	Naïve	2	02AG/F2
17	NYU6556‐3	M	34	February 2008	February 2001	174	17,742	Naïve	2	02AG/G
18	NYU6541‐6	F	31	November 2004	July 2002	169	65,554	Naïve	1	02AG/F2

NA, not available; NFGS, near full‐genome sequencing; MDS, medical diagnostic center.

**Figure 1:**
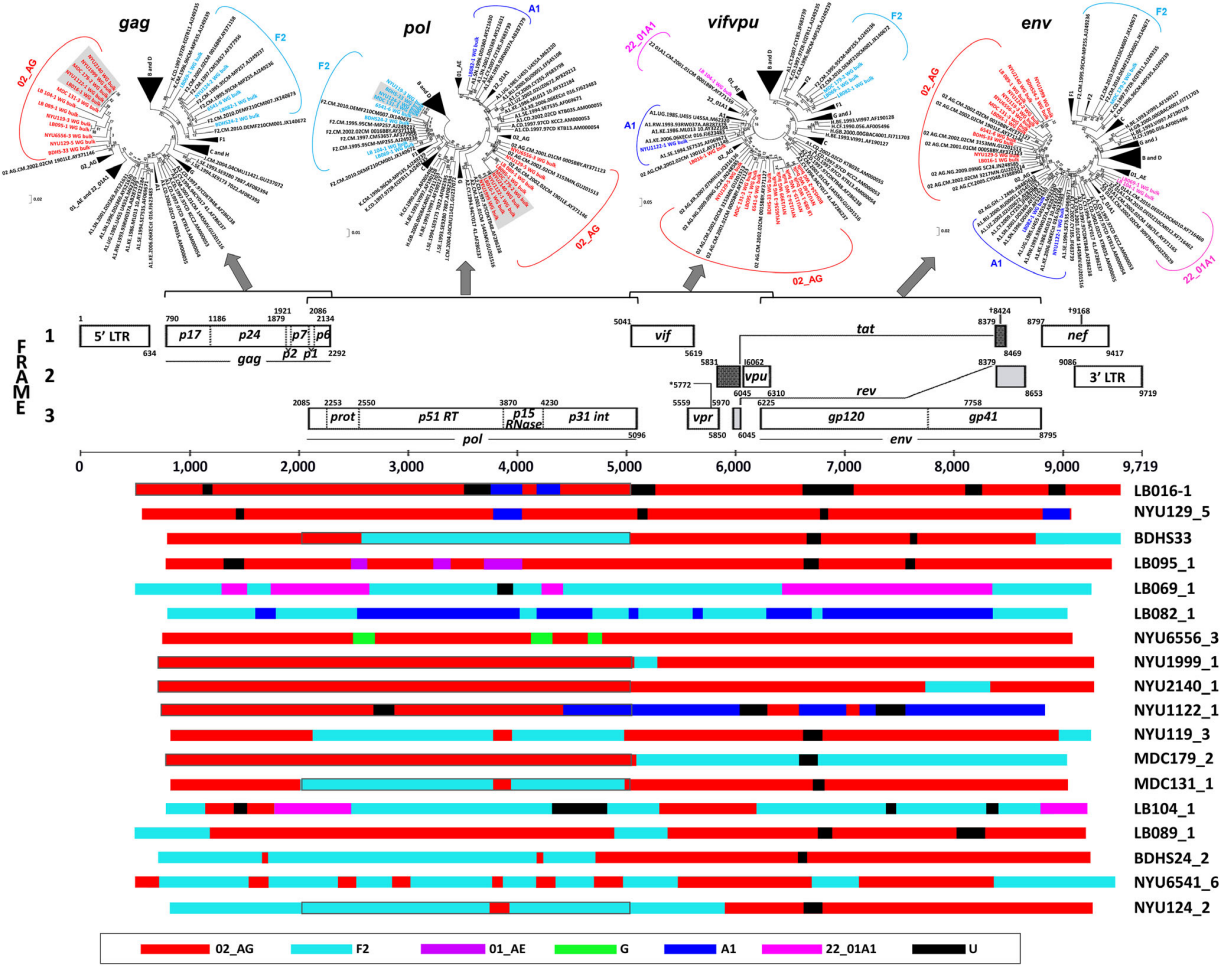
Recombinant breakpoint patterns of URFs from Cameroon over the near full genome and for selected sub‐regions (**A**) Maximum Likelihood phylogenetic trees of separate genomic regions (*gag, pol, vif, vpr, vpu* and *env*) of the near full genome sequences (NFGS), generated using MEGA5.2. Reference sequences are shown in black (LANL database); study sequences are coloured according to subtype. Brackets enclose all sequences per subtype. (**B**) Schematic representation of the mosaic composition of the 18 studied NFGS. The HxB2 genome map (GenBank: K03455) is shown for genomic orientation. The legend at the bottom indicates the colour code for subtype representation. Genetic distances <0.2%, as observed in *gag* and *pol* trees, are marked with grey background in the trees and with grey surrounding boxes in the schematic. NFGS, near full genome sequencing.

Sanger NFGS was obtained for all 18 URF samples (Figure 1, Figures S1, S2 and Table 1), complemented by selective 3GS, SGA and cloning experiments (see also Banin *et al*. 31). The URF near full genomes were composed of the pure (sub‐)subtypes A (A1), F (F2), and G as well as the CRFs CRF01_AE, CRF02_AG and CRF22_01A1. The recombinant breakpoints were scattered along the full genome; among the 18 URF near full genomes, no recurrent breakpoint positions became evident. However, comparing the URFs according to functional genomic units, it was apparent that CRF02_AG was dominant in *gag*, accessory genes (*vif, vpr, vpu*), and *env*, whereas F2 had high abundance in *pol* (Figure 1). Though small in size, notably more than 70% (13/18) of the studied URFs exhibited undefined regions, which could not be attributed to any pure subtype or currently known CRF (a common nonexclusive selection of reference strains is shown in Figure S1). The most complex mosaic pattern was found for LB104‐1, being composed of F2, CRF_02AG, CRF_2201A1 and undefined regions. In the highly conserved *gag* and *pol* regions, repeated analyses yielded low genetic distances (<0.2%) between a few samples. Follow‐up studies with longitudinal samples will determine whether genetic linkages within the cohort or impurities during sample processing accounted for this observation (Figure 1, grey highlighted regions).

### 3.2 Common phylogenetic background patterns in Cameroonian URFs

**Figure 2:**
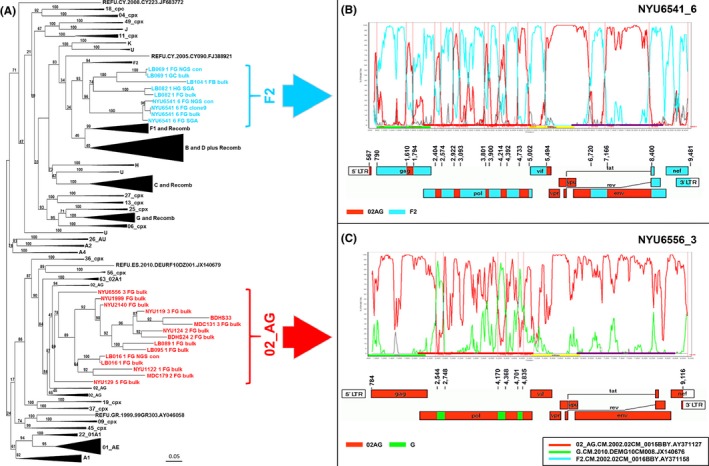
Near full genome phylogenetic analysis reveals two common subtype background patterns in URFs from Cameroon (**A**) Maximum likelihood tree of NFGS (HxB2 pos 596 to 9542) including study sequences in the indicated colour code and subtype and circulating recombinant form reference sequences (72 Reference panel, LANL database) in black. Brackets enclose all study sequences per subtype. Large branches of reference sequences that are distant from the study sequences were collapsed for clarity. URFs that clustered with CRF02_AG reference sequences are shown in red (CRF02_AG backbone) and those that cluster with subtype F2 are shown in cyan (F2 backbone). (**B** and **C**) One representative NFGS is shown for each cluster, that is, NYU6541_6 for F2 (**B**) and NYU6556_3 for CRF02_AG (**C**). Bootscan plots (Simplot) are shown in the upper panel and schematic representations of the breakpoint patterns (Recombinant Drawing tool, LANL database) in the lower panel. Reference strains used for Simplot analyses are boxed. NFGS, near full genome sequencing.

Phylogenetic breakpoint analysis revealed a heavy presence of mosaic CRF02_AG and F2 segments in the studied URFs (Figure 1, Figures S2, S3, Table S1). Interestingly, and following the segregated breakpoint analysis, two common clustering patterns of the URFs were identified when phylogenetic analyses were performed using the near full genomes, i.e. URFs either clustered with CRF02_AG or F2 clades (Figure 2). The CRF02_AG clustering was predominant and applied to fourteen URFs (NYU6556‐3, NYU1999‐1, NYU2140‐1, NYU119‐3, BDHS33, MDC131‐3, NYU124‐2, BDHS24‐2, LB089‐1, LB095‐1, LB016‐1, NYU1122‐1, MDC179‐2 and NYU129‐5) (Figure 2, red). Nested within the F2 branch, four URFs (LB069‐1, LB104‐1, LB082‐1 and NYU6541‐1) were identified (Figure 2, cyan). Of note, seven more URFs were composed by more than a third of their near full genomes of F2 sequences (Figure 1, Figures S2, S3). F2 regions were most abundant in *pol*, followed by *gag* and the border region between the end of *env* and *nef*. F2 most frequently recombined with CRF02_AG, A1, CRF22_01A1 and undefined regions. Overall, dominant CRF02_AG and F2 framework regions appeared as a common pattern in all of the studied URFs from Cameroon.

### 3.3 High degree of intra‐patient URF diversity determined by 3GS

**Figure 3:**
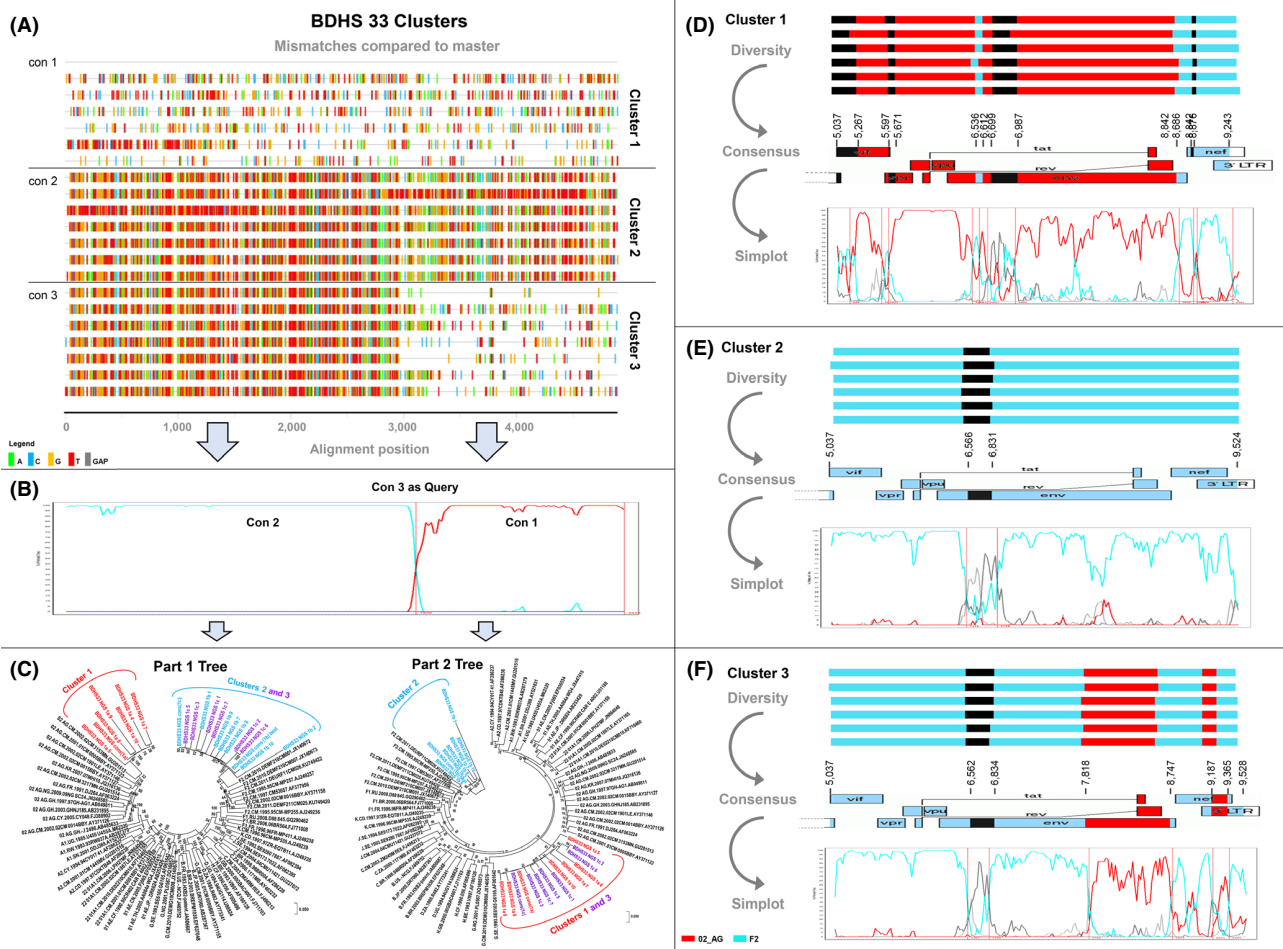
Intra‐patient URF diversity in subject BDHS‐33 determined by deep sequencing analysis (**A**) Highlighter plot of a selection of 18 representative third‐generation sequencing (3GS) reads with three consensus sequences (con 1 to con 3) according to the identified clusters (1 to 3). Mismatches compared to con 1 as master sequence are shown as bars coloured according to the legend. The sequence reads are partitioned according to the identified clusters, and the vertical red line indicates the internal breakpoint dividing part 1 (F2‐like) and part 2 (02‐AG like). (**B**) Bootscan analysis of intra‐patient recombinants was done using con 3 as query sequence and con 1 and con 2 as reference sequences (SimPlot, window size 200, step size 20). (**C**) Separate maximum likelihood phylogenetic trees for each recombinant genomic sub‐region (parts 1 and 2) as determined in (**A** and **B**). Reference sequences are shown in black (LANL database); study sequences are coloured according to clusters. Brackets enclose all study sequences per subtype (F2 in cyan, CRF02_AG in red). (**D**,** E** and **F**) Schematic illustration of viral diversity between and within clusters 1, 2 and 3. For each cluster, recombination schematics are shown for six representative reads (upper panel) and the derived consensus sequence (middle panel), indicated in the form of a genomic map (Recombinant drawing tool, LANL database). Bootscan plots (Simplot) of the consensus sequences are shown in the lower panel. Relative abundance of each viral sub‐populations (cluster) as determined by 3GS is shown in brackets (%).

Twelve samples were subjected to 3GS analyses using the portable Minion technology suited for long read lengths. 3GS enabled to determine the extent of intra‐patient URF diversity and evolution. According to the differential polymerase chain reaction (PCR) amplification efficiencies of the near full genomes, HGs or shorter constructs per subject (Table 1) 31, we performed 3GS with a selection of three near full genomes, six HGs, and three *vif/gp120* products. Based on the limited number of 3GS amplification rounds (15×) (to minimize amplification artefacts), the long read lengths, and the intrinsic limitations in yield using the recently introduced portable 3GS technology, the depth of 3GS remained low to moderate with mostly few thousand HIV‐specific reads per sample (Figure S4) 31. After removal of singular reads and possible contaminants, the long reads were subjected to phylogenetic analyses including maximum likelihood trees, highlighter and breakpoint analysis. Comparing the outcomes of 3GS with single‐genome amplification and bulk sequencing confirmed genetic similarities and comparable breakpoint patterns thus, the suitability of our 3GS approach 31. Three participants were identified with a remarkable diversity of viral populations simultaneously present in plasma, that is, BDHS33, NYU2140‐1 and NYU124‐2 (Figure 3, Figures S5, S6). The other studied 3GS samples revealed homogeneous viral populations with consistent breakpoint patterns, as shown representatively for four participants (Figure S7). A high intra‐patient diversity was observed for subject BDHS33, studied over the second HG (Figure 3A,B,C,D,E,F). Three viral sub‐populations were identified, which genetically differed by 6% to 17%. According to the 3GS results, each sub‐population made up > 2% of the entire viral population (Figure 3D,E,F), which minimized the likelihood that *in vitro* recombination was the sole cause for the presence of the detected recombinants 31. All three sub‐populations were identified as recombinant viruses composed of CRF02_AG, F2, and undefined regions. While two URF sub‐populations were genetically unrelated (clusters 1 and 2), the third URF sub‐population (cluster 3) was composed of sequences of the other two sub‐populations (parts 1 and 2 respectively; Figure 3A,B,C) suggesting the co‐presence of a secondary URF together with its parental URF lineages. Besides BDHS33, subjects NYU2140‐1 and NYU124‐2 exhibited high intra‐sample HIV‐1 diversities with four unique viral sub‐populations (clusters a to d) (Figures S5, S6). In each sample, either one (cluster b to 75%) or two populations (cluster b to 45% and cluster c to 31%) predominated respectively. Bulk amplification with Sanger sequencing selectively detected one of the 3GS sub‐populations with >6% prevalence, which was the major population for BDHS33 (cluster 1, 52%), but not for NYU124‐2 (cluster a, 19%) and NYU2140‐1 (cluster c, 7%).

### 3.4 DRM, co‐receptor tropism and Env epitope analysis in URFs from Cameroon

To assess whether the URFs potentially inherited impaired sensitivities to antiretroviral treatment, the entire *pol* regions were studied for mutations conferring resistance to protease inhibitors (PI), reverse transcriptase inhibitors (RTI), and integrase inhibitors (INSTI) (Table 2). The bulk sequences of the 18 participants were subjected to a genotypic DRM analysis using the Stanford and WHO HIV drug resistance databases. Five out of 18 subjects exhibited accessory DRMs including mutations V32E (PI), T97A and Q146H (both INSTI). T97A was most frequently observed (4×). No canonical RTI or major PI or INSTI resistance mutation was detected. Genotypic co‐receptor analysis, using Geno2pheno and the settings recommended for CRF02_AG infections 43 predicted exclusive CCR5 usage for all studied URFs, whereas PhenoSeq, using settings optimized for subtype A and CRF02_AG, predicted CXCR4 tropism for 4/18 URF viruses (Table 2). To analyse the URFs’ immunological features and putative resistance mutations against broadly neutralizing antibodies (bnAbs), envelope protein (Env) amino acid alignments of functional URF bulk sequences were studied (Figure S8). As expected, the URFs exhibited highly diverse variable regions V1‐V5, in contrast to the constant regions, which were mostly consistent in length and amino acid composition. Potential N‐glycosylation sites with relevance for bnAbs were highly conserved for most sites (≤1 out of 17 mutated); however, frequent resistance mutations were observed at sites N234 (8/17) and N637 (3/17). Of interest, both sites are relevant for gp120/gp41 interphase bnAbs, such as 8ANC195 and PGT151 48, 49. Also, sites of immune pressure identified in the human RV144 vaccine trial 45, 46 (4 to 5 deviating residues per site) and CD4 binding site (CD4bs) residues capable of conferring resistance to CD4bs bnAbs 47 (up to twelve deviating residues per site) showed greater divergence (Figure S8).

**Table 2:** HIV drug resistance (HIVDR) mutations and genotypic co‐receptor usage analysis of the studied URF near full genomes

No.	Sample ID	PI Resistance Mutations		RTI Resistance Mutations		INSTI Resistance Mutations		Co‐receptor tropism	
		Major	Accessory	NRTI	NNRTI	Major	Accessory	G2P (5% FPR)	PhenoSeq
1	LB016‐1	None	None	None	None	None	None	R5	R5
2	LB069‐1	None	None	None	None	None	None	R5	R5
3	LB082‐1	None	None	None	None	None	None	R5	R5
4	LB089‐1	None	None	None	None	None	None	R5	**X4**
5	LB095‐1	None	None	None	None	None	None	R5	R5
6	LB104‐1	None	None	None	None	None	**T97A**	R5	**X4**
7	MDC131‐3	None	None	None	None	None	None	R5	R5
8	MDC179‐2	None	None	None	None	None	None	R5	**X4**
9	BDHS24‐2	None	**V32E**	NA	NA	None	**T97A**	R5	R5
10	BDHS33	None	None	None	None	None	None	R5	**X4**
11	NYU119‐3	None	None	None	None	None	None	R5	R5
12	NYU124‐2	None	None	None	None	None	None	R5	R5
13	NYU129‐5	None	None	None	None	None	**T97A**	R5	**X4**
14	NYU1122‐1	None	None	None	None	None	**Q146H**	R5	R5
15	NYU1999	None	None	None	None	None	None	R5	R5
16	NYU2140‐1	None	None	None	None	None	None	R5	R5
17	NYU6556‐3	None	None	None	None	None	None	R5	R5
18	NYU6541‐6	None	None	None	None	None	**T97A**	R5	R5

The presence of drug resistance mutations, according to the Stanford and WHO drug resistance database and X4 tropism are highlighted in bold. FPR, false positive rate; G2P, Geno2Pheno; INSTI, integrase strand transfer inhibitor; NA, Not applicable due to a frameshift in the respective genomic region; NNRTI, non‐NRTI; NRTI, nucleoside reverse transcriptase inhibitor; PI, protease inhibitor; R5, predicted CCR5 tropism; RTI, reverse transcriptase inhibitor; URF, unique recombinant form.; X4, predicted CXCR4 tropism.

## 4 Discussion

Eighteen URFs that were identified between the years 2000 and 2015 in the HIV recombinant hotspot Cameroon were characterized using a newly established NFG amplification and sequencing protocol 31. The study provided insight into the evolving HIV‐1 diversity, the genomic complexity of URF infections, the structural dynamics of emerging recombinant forms, and possible implications/caveats for antiretroviral treatment and vaccine research.

The URFs exhibited divergent recombination patterns with mosaic pieces of diverse subtypes and CRFs scattered along the whole genomes. It underlined the necessity of performing NFGS in order to achieve accurate HIV surveillance, subtyping and genotypic analyses. The URFs were composed of pure (sub‐)subtypes A (A1), F (F2) and G, and recombinant forms CRF02_AG, CRF01_AE and CRF22_01A1, thus, URFs appeared to have taken up key circulating strains in Cameroon 12, 13, 17, 50. Notably, 16/18 URFs contained CRF02_AG sequences and 13/18 URFs contained F2 sequences. For all URFs, the frameworks were exclusively built of CRF02_AG or F2 sequences. While CRF02_AG has been well documented to be the predominant lineage and the major fundament of recombinant strains in Cameroon 11, 12, 18, 24, 51, F2 is less known, and the growing influence of F2 in the URF genetic pool only recently became evident 12, 13. Within the last two decades, F2 infections have steadily increased in Cameroon and spread beyond the Cameroonian borders in West and Central Africa 12, 13, 17, 52, 53, 54, 55, 56, which likely promoted the involvement of F2 sequences in emerging recombinant forms. A CRF is declared when a characteristic recombination pattern (of a former URF) recurs in at least three epidemiologically unlinked individuals. While CRFs composed of CRF02_AG and F2 have not been described so far, the current and recent studies mentioned above suggest their likely presence and a timely detection when a higher number of URF samples is analysed.

Although bulk sequencing covered a large part of the relevant information in terms of strain monitoring, deep sequencing provided more profound insight into strain diversity and presence of subdominant subtypes/recombinants within an individual. Whereas bulk sequencing identifies one (or a mix of few variants) in a sample, which is dominant in terms of relative abundance or amplification yield, deep sequencing can detect several co‐existing variants. It enables the identification of otherwise undetectable variants/recombinants and may reveal the presence of (recombination events with) additional subtypes. For example, Figure 3, Figures S5, and S6 showed that multiple strains were present in three individuals at the studied time points, which bulk sequencing could not identify. Furthermore, bulk sequencing almost exclusively detected CRF02_AG in the 3’ HG of #NYU124_2 viruses (Figure 1), which was comparable to cluster a of the deep sequencing results (Figure S6). In contrast, deep sequencing revealed the presence of three additional viral populations, of which two were dominated by F2 sequences (along the studied 3’ half of the genome; clusters b and d in Figure S6).

Irrespective of sequencing method, a one amplicon strategy, as successfully applied for 7/18 samples, was the preferable NFGS approach since the assembly of multiple amplicons bears the potential risk of obtaining chimeras composed of different variants circulating in the same subject. The likeliness to obtain chimeras increases with the number of composite amplicons. The considerable size of the overlapping regions in our multiple amplicon approaches (e.g. >900 bp in two‐amplicon approaches) helped to avoid artificial chimeras. Assembly inaccuracies cannot be entirely excluded when using multiple partial amplicons (≥2). Yet, deep sequencing in combination with assembly algorithms exist for the correct assembly of large composite genomes, thus, to minimize assembly artefacts 57, 58.

The detection of numerous undefined regions in 2/3 of the studied URFs implies that the genetic pool of HIV is still insufficiently explored. Plausible reasons are evolutionary leaps of the circulating strains or the presence of unidentified strains/clades including traces of ancient viruses 23. Since the undefined regions were rather small in size (<500 bp), a lack of strong phylogenetic signals might have contributed that some fragments remained undefined. Besides HIV's high intrinsic evolutionary rate due to its error‐prone replication process, intra‐ or inter‐subtype recombination expedites its rapid evolution. Recombination results from super‐ or dual infection of an individual with two or more genetically distinct viruses 21, 59. The detected high intra‐patient diversity in three out of 11 successfully determined 3GS samples indicated that the diversification of viral strains through recombination was actively ongoing. Of interest, one participant exhibited three URF populations including a secondary URF and both of its parental URF lineages that most likely served as genetic templates after the occurrence of dual infection. Although a thorough confirmation of each 3GS sub‐population through extensive SGA analyses was beyond the capacity and scope of the current study, the SGA validation of our 3GS approach and the exclusion of minority variants with less than 2% prevalence makes it appear likely that *in vitro* recombination was not the only source for the detected secondary URF 31, 60, 61. Further studies are needed to retrace the events of *in vivo* recombination fully and to assess the clinical and functional consequences of high URF diversity within each individual and the entire population.

Subtype‐specific differences in replicative fitness have been described, and in the case of URFs, replication kinetics were associated with subtype characteristics of the *pol* segment 11. Specifically, *in vitro* replication was enhanced in URFs with *pol* regions of clades CRF01_AE, CRF22_01A1 or D when compared with CRF02_AG. Of interest, our study showed overall predominant CRF02_AG sequences; however, not for the *pol* regions where a strong influence of other clades, specifically F2, became evident. Recent recombinant pattern analysis revealed that, on a global scale, subtype F fragments most frequently contributed to recombinants in *gag*,* protease*,* reverse transcriptase*,* integrase* and gp120 genomic regions, confirming the tendency of subtype F to recombine in *pol* 62. The focus on Cameroon, the predominance of sub‐subtype F2 over F1, and the limited number of 18 NFGS in our study may be responsible for the subtle differences in F fragment distribution compared with the global study. Overall, these data suggest that the generation of new URFs and onward transmission may be partly driven by replication characteristics of the parental strain *pol* regions.

The *pol* region is also the primary target for antiretroviral therapy, and sequence evolution in *pol* is dependent on drug selection pressure. Controversially discussed, polymorphisms known to confer drug resistance may generally occur more frequently in individuals/populations with the higher genetic diversity of HIV strains 63, 64. Our study included ART‐naïve individuals with no indications of treatment history, in whom no signs of major canonical DRMs were detected. However, 5/18 individuals were identified to carry accessory DRMs, which, in the context of the mosaic genetic composition, might confer new ways of drug resistance. Notably, while the emerging subtype F2 strains are largely understudied, the genetically closely related F1 viruses were shown to exhibit reduced sensitivities to *reverse transcriptase* and *protease* inhibitors 65, 66.

More generally, higher pre‐treatment diversity has been associated with higher viral load, less effective viral control after treatment interruption, accelerated disease progression 67, 68, 69, and impact on drug efficacy while under treatment 63, 70, 71. Presumably, an expedited process of escape is provided by an outgrowth of resistant variants or recombination events out of a larger pool of available strains. The presence of multiple viral sub‐populations in 3/11 study samples that were subjected to 3GS suggests that such processes are of relevance in our study population. More precise deep sequencing as obtained with the portable MinIon 3GS technology, with the given limitations in depth and accuracy 31, 72, 73, will be of importance to define the clinical consequences of high intra‐patient URF diversity.

With regards to the antibody‐exposed Env regions of the studied URF viruses, sites of immune pressure, as identified in the human RV144 vaccine trial 45, 46 varied in half of the studied URFs. Also, essential residues for binding/neutralization of gp120/gp41 interphase as well as conformational CD4 binding site bnAbs were divergent in more than half of the studied strains 47, 48, 49. It indicates that differential quaternary Env arrangements and adaptations may have evolved/been necessary upon the generation of novel URFs. Longitudinal follow‐up of individuals with URF infections including clinical and immunological monitoring will be paramount to comprehensively assess and counteract the challenges URFs pose for human healthcare.

## 5 Conclusions

The current study illustrated the genetic complexity and structural dynamics in URFs from Cameroon. Periodic monitoring of emerging strains in Cameroon and other regions in West‐Central Africa is necessary to detect evolutionary trends that might appear in other regions of the world at a later time, as monitored in the past for the migration of major HIV‐1 M subtypes. The complications of diverse HIV infections may, among other factors, contribute to regional public healthcare issues as evident in the most recent 90‐90‐90 target report from UNAIDS; West and Central Africa had one of the most pronounced treatment target gaps in the world, i.e. percentages below 50% in diagnosed, treated and virally suppressed people living with HIV. Ongoing full genome monitoring of increased numbers of URF infections will reveal, whether the dominant CRF02_AG and F2 framework patterns will continue to persist or further evolve, and how the challenges that emerging URFs pose for HIV diagnosis, antiretroviral treatment and vaccine development can be addressed.

## Competing interests

The authors have no competing interests to declare.

## Authors’ contributions

RD designed the study. ANB, MTu, JSB, PZ, IOO and XW performed experiments. ANB, MTu, MTo, AKJ and RD performed experimental and statistical data analysis. AJN, DM, JN, MKG, AH and CF supervised experimental and statistical procedures. ANB and RD wrote the manuscript. All authors reviewed the manuscript.

## Supporting information


**Figure S1.** Similarity and Bootscan analyses for the determination of near full genome recombinant breakpoint patterns. (**A**) Similarity plots were done using reference sequences of most common pure subtypes and circulating recombinant forms (CRFs) to identify the subtype composition of the studied URFs (shown for BDHS‐33). (**B**) BootScan plots were done using the best matching reference subtypes as identified in the Similarity plots (**A**) and two outlier reference sequences (here: clade B and D reference strains). Vertical red lines indicate recombination breakpoints. Y‐axis indicates sequence similarity (**A**) or bootstrap values (**B**). The x‐axis covers the studied near full genome region. Standard settings were used: window size 200, step size 20, 250 replicates. Reference sequences used for Simplot analyses are boxed.
**Figure S2.** Recombinant breakpoint analysis of 18 Cameroonian URFs. URFs using Simplot. BootScan breakpoint analysis of the 18 studied NFGS determined by bulk sequencing (Simplot, window size 200, step size 20) using the indicated reference sequences (boxed). A schematic representation of the URFs is shown below each Simplot analysis (Recombinant Drawing tool, LANL database). The subtype colour codes are indicated in the lower left, respectively.
**Figure S3.** Phylogenetic analysis of recombinant fragments. Maximum likelihood tree indicating the phylogenetic relationship between recombinant fragments >900 bp from the 18 studied bulk NFGS (60 fragments in total) together with full genome reference sequences downloaded from the LANL database and GenBank representing the broad HIV‐1 group M diversity 1. The un‐rooted tree was constructed with 1000 bootstrap replicates using RAxML version 8 2. Some clades have been condensed for the sake of clarity. Fragments of the study samples are coloured per subject according to the colour code on the upper right; reference sequences are shown in black. Recombinant fragments are numbered according to their appearance in the NFGS from 5’ to 3’ and as shown in Figure 1 and Figure S2. The findings of the RAxML‐based phylogenetic subtype classification and comparisons with Simplot results are summarized in Table S1.
**Figure S4.** Read length distribution of URF third‐generation sequencing. Left: Dot and box‐whisker plot indicating read lengths in base pairs (y‐axis) of third‐generation sequencing (3GS) for eleven URF samples (x‐axis). Grey dots represent individual reads. Coloured boxes display the middle 50% of data points. Medians are shown as horizontal lines within the boxes. Whiskers indicate variability outside the upper and lower quartiles. Right: Bar plot showing mean read lengths and standard deviations (y‐axis) for the eleven 3GS study samples.
**Figure S5.** Intra‐patient URF diversity in NYU2140_1. (**A**) Highlighter plot of 24 representative 3GS reads and four consensus sequences (con a to con d) according to the four identified sequence clusters (a to d), determined for the second half genome (HxB2 position 5037‐9555). Mismatches compared to the master sequence con a are shown as coloured bands according to the legend. The sequence reads are partitioned according to the identified clusters and separated by a grey line. Relative abundance of each viral sub‐populations (cluster) as determined by 3GS is shown in brackets (%). (**B**,** C**,** D** and **E**) Schematic illustration of recombinant strain diversity between and within clusters a, b, c and d. For each cluster, six representative reads (upper panel) and the respective consensus sequence (middle panel, done with Recombinant drawing tool) are shown. Bootscan plots of the consensus sequences are shown in the lower panel. 3GS: Third‐generation sequencing.
**Figure S6.** Intra‐patient URF diversity in NYU124‐2. (**A**) Highlighter plot of 24 representative 3GS reads and four consensus sequences (con a to con d) according to the four identified sequence clusters (a to d), determined for the *vif/gp120* position (HxB2 position 4956‐7838). Mismatches compared to con a as master sequence are shown as coloured bands according to the legend. The sequence reads are partitioned according to the identified clusters and separated by a grey line. Relative abundance of each viral sub‐population (cluster) as determined by 3GS is shown in brackets (%). (**B**,** C**,** D** and **E**) Schematic illustration of recombinant strain diversity between and within clusters a, b, c and d. For each cluster, six representative reads (upper panel) and the respective consensus sequence (middle panel, done with Recombinant drawing tool) are shown. Bootscan plots of the consensus sequences are shown in the lower panel. 3GS: Third‐generation sequencing.
**Figure S7.** Intra‐patient URF diversity in NYU6541_6, MDC179‐2, LB069_1 and NYU1122_1. Schematic illustration of recombinant strain diversity within subjects, determined for the near full genomes of NYU6541_6 and LB069_1, and for the *vif/gp120* genomic regions of MDC172‐2 and NYU1122‐1. Six representative 3GS reads (upper panel) and the respective consensus sequence (middle panel, done with Recombinant drawing tool) are shown per subject. 3GS: Third‐generation sequencing.
**Figure S8.** Env Amino acid alignment with indicated N‐glycosylation sites, bnAb epitopes and sites of immune pressure. Amino acid alignment (Clustal Omega) of functional Env sequences from 17 bulk‐amplified URFs (LB089‐1 not included because of a frame shift in Env) in comparison to subtype B (HxB2), CRF02_AG (0014BBY) and F2 (CM53657) reference sequences. Numbering of amino acid residues is based on HxB2 Env. N‐glycosylation sites are highlighted in red (N‐glycosite tool of the LANL database). N‐glycosylation sites critical for selected bnAbs are boxed in red with yellow background: N88 (gp120/gp41 interphase bnAb 35O22), N156 and N160 (V2 glycan bnAbs, e.g. PG9/PG16), N234 and N276 (gp120/gp41 interphase bnAb 8ANC195), N301 and N332/334 (V3 glycan bnAbs, e.g. PGT121/PGT128) as well as N611 and N637 (gp120/gp41 interphase bnAb PGT151). Sites of immune pressure in the RV144 vaccine trial [K169, V172, or mismatch (mm) at I181] and sites of resistance to CD4bs bnAbs are boxed in green and blue respectively, according to deCamp *et al*. 3, Rolland *et al*. 4, and Courtney *et al*. 5. Deviant/resistance conferring residues are highlighted with a red box.
**Table S1.** Comparison of phylogenetic and Simplot‐based subtype classifications of recombinant fragments.
