# Supplementary material for: Near full genome characterization of HIV‐1 unique recombinant forms in Cameroon reveals dominant CRF02_AG and F2 recombination patterns
Source: J Int AIDS Soc. 2019 Jul 28;22(7):e25362. doi: 10.1002/jia2.25362 (PMC6661401; doi:10.1002/jia2.25362)
Supplement: Supplementary file 1 — Figure S1. Similarity and Bootscan analyses for the determination of near full genome recombinant breakpoint patterns. (A) Similarity plots were done using reference sequences of most common pure subtypes and circulating recombinant forms (CRFs) to identify the subtype composition of the studied URFs (shown for BDHS‐33). (B) BootScan plots were done using the best matching reference subtypes as identified in the Similarity plots (A) and two outlier reference sequences (here: clade B and D reference strains). Vertical red lines indicate recombination breakpoints. Y‐axis indicates sequence similarity (A) or bootstrap values (B). The x‐axis covers the studied near full genome region. Standard settings were used: window size 200, step size 20, 250 replicates. Reference sequences used for Simplot analyses are boxed. Figure S2. Recombinant breakpoint analysis of 18 Cameroonian URFs. URFs using Simplot. BootScan breakpoint analysis of the 18 studied NFGS determined by bulk sequencing (Simplot, window size 200, step size 20) using the indicated reference sequences (boxed). A schematic representation of the URFs is shown below each Simplot analysis (Recombinant Drawing tool, LANL database). The subtype colour codes are indicated in the lower left, respectively. Figure S3. Phylogenetic analysis of recombinant fragments. Maximum likelihood tree indicating the phylogenetic relationship between recombinant fragments >900 bp from the 18 studied bulk NFGS (60 fragments in total) together with full genome reference sequences downloaded from the LANL database and GenBank representing the broad HIV‐1 group M diversity 1. The un‐rooted tree was constructed with 1000 bootstrap replicates using RAxML version 8 2. Some clades have been condensed for the sake of clarity. Fragments of the study samples are coloured per subject according to the colour code on the upper right; reference sequences are shown in black. Recombinant fragments are numbered according to their appearance in the NFGS [file JIA2-22-e25362-s001.docx]

**Supporting Information**

Manuscript: “Near Full Genome Characterization of HIV-1 Unique Recombinant Forms in Cameroon reveals dominant CRF02_AG and F2 recombination patterns.” A.N. Banin, *et al*


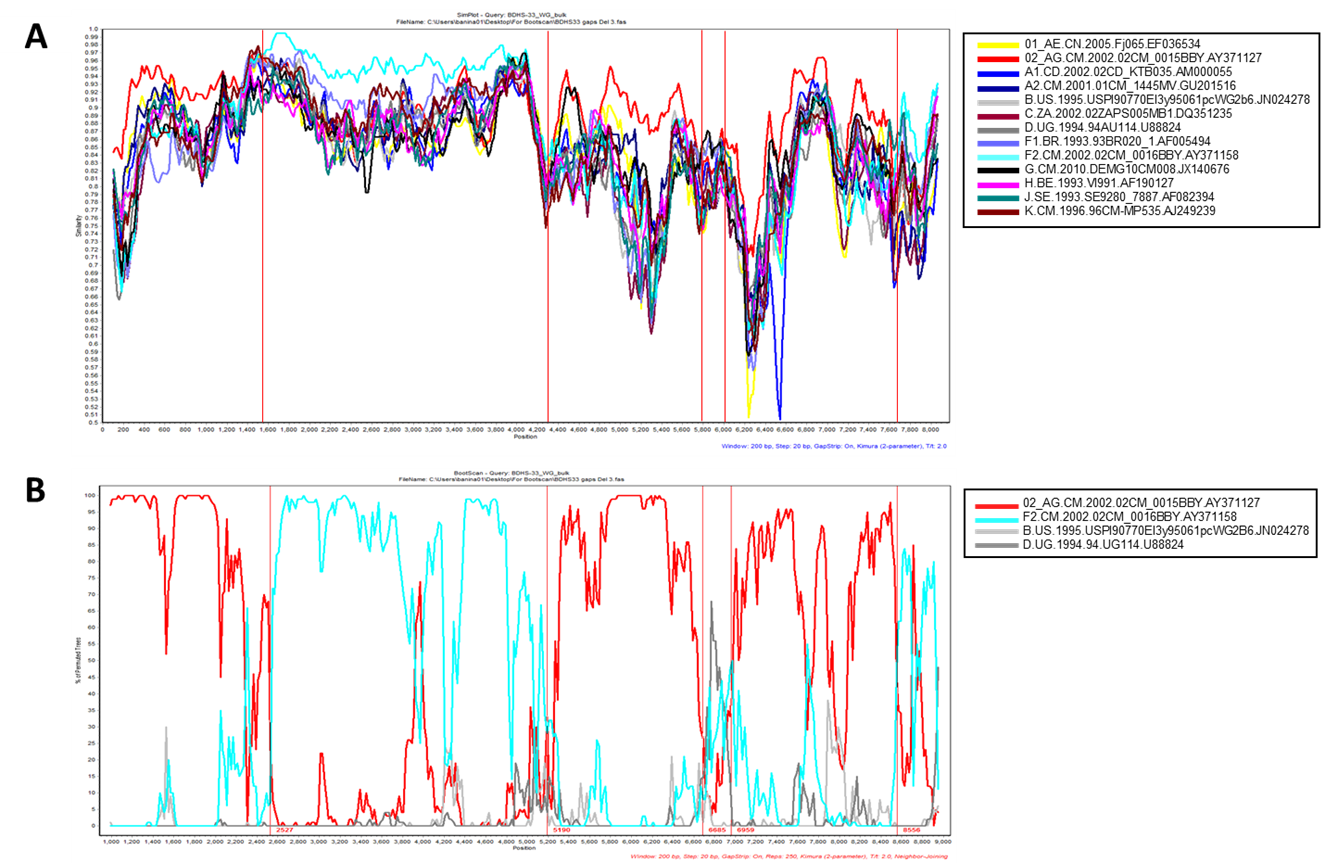


**Supplemental Figure 1: Similarity and Bootscan analyses for the determination of near full genome recombinant breakpoint patterns: A)** Similarity plots were done using reference sequences of most common pure subtypes and circulating recombinant forms (CRFs) to identify the subtype composition of the studied URFs (shown for BDHS-33). **(B)** BootScan plots were done using the best matching reference subtypes as identified in the Similarity plots **(A)** and two outlier reference sequences (here: clade B and D reference strains)**.** Vertical red lines indicate recombination breakpoints. Y-axis indicates sequence similarity **(A)** or bootstrap values **(B)**. The x-axis covers the studied near full genome region. Standard settings were used: window size 200, step size 20, 250 replicates. Reference sequences used for Simplot analyses are boxed.

**Supplemental Figure 2. Recombinant breakpoint analysis of 18 Cameroonian URFs using Simplot.** BootScan breakpoint analysis of the 18 studied NFGS determined by bulk sequencing (Simplot, window size 200, step size 20) using the indicated reference sequences (boxed). A schematic representation of the URFs is shown below each Simplot analysis (Recombinant Drawing tool, LANL database). The subtype color codes are indicated in the lower left, respectively.


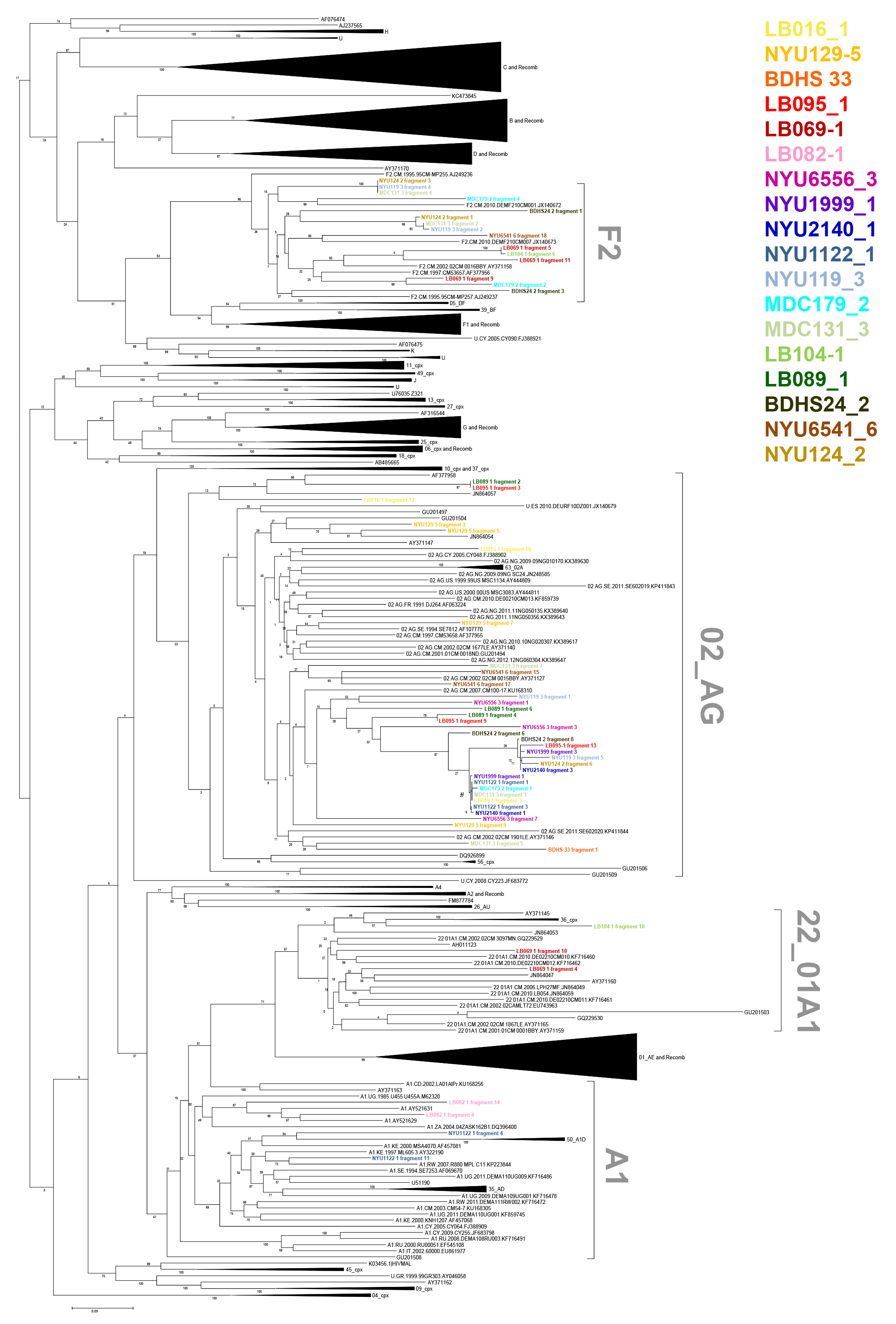


**Supplemental Figure 3. Phylogenetic analysis of recombinant fragments.** Maximum likelihood tree indicating the phylogenetic relationship between recombinant fragments >900 bp from the 18 studied bulk NFGS (60 fragments in total) together with full genome reference sequences downloaded from the LANL database and GenBank representing the broad HIV-1 group M diversity [1]. The un-rooted tree was constructed with 1,000 bootstrap replicates using RAxML version 8 [2]. Some clades have been condensed for the sake of clarity. Fragments of the study samples are colored per subject according to the color code on the upper right; reference sequences are shown in black. Recombinant fragments are numbered according to their appearance in the NFGS from 5’ to 3’ and as shown in **Figures 1 and S2**. The findings of the RAxML-based phylogenetic subtype classification and comparisons with Simplot results are summarized in **Table S1**.


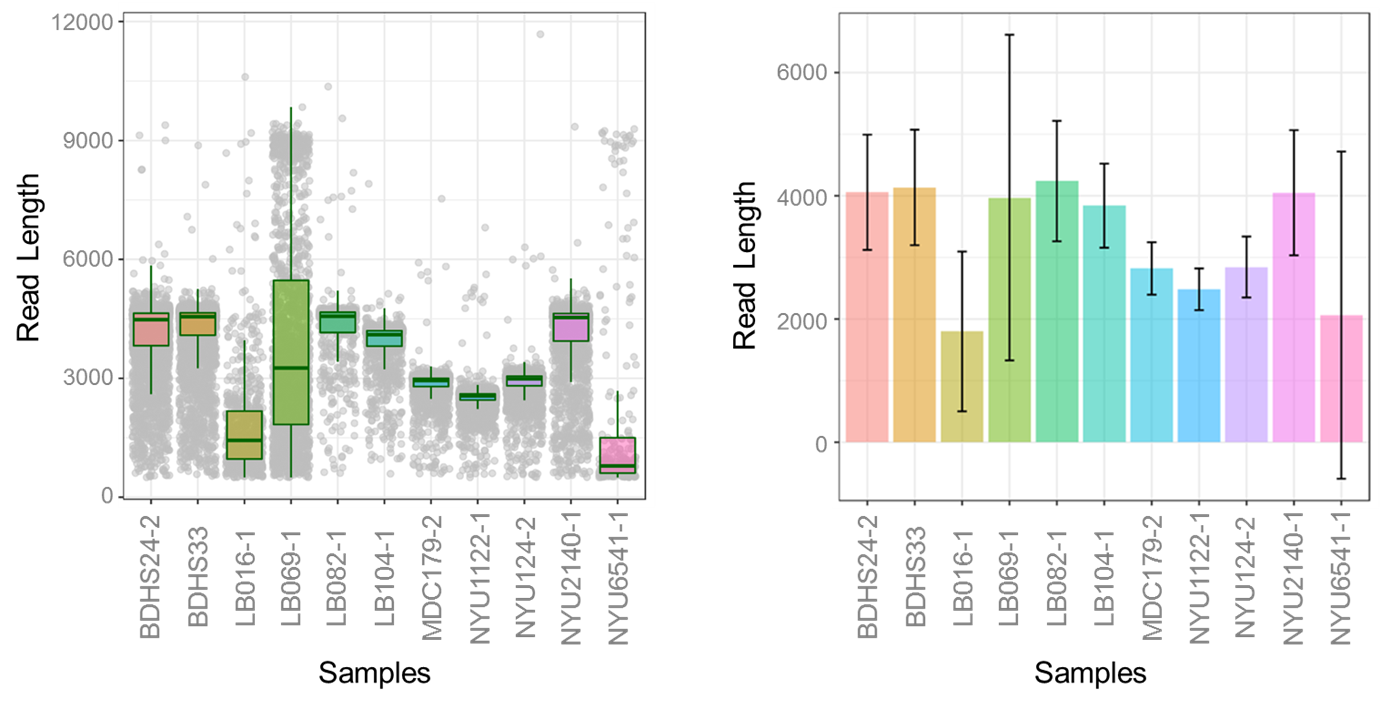


**Supplemental Figure 4. Read length distribution of URF third-generation sequencing. Left:** Dot and box-whisker plot indicating read lengths in base pairs (y-axis) of third-generation sequencing (3GS) for eleven URF samples (x-axis). Gray dots represent individual reads. Colored boxes display the middle 50% of data points. Medians are shown as horizontal lines within the boxes. Whiskers indicate variability outside the upper and lower quartiles. **Right**: Bar plot showing mean read lengths and standard deviations (y-axis) for the eleven 3GS study samples.


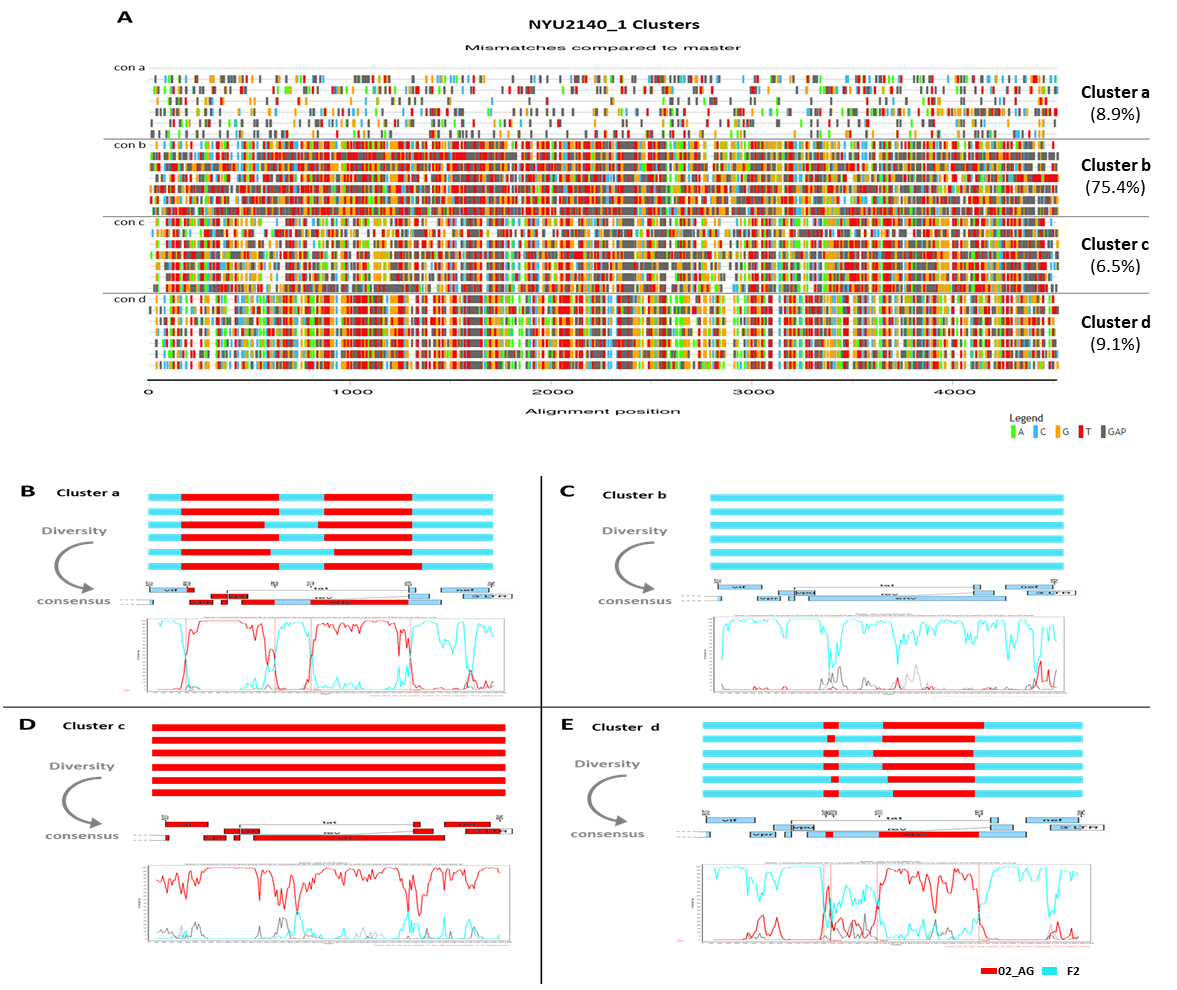


**Supplemental Figure 5. Intra-patient URF diversity in NYU2140_1. (A)** Highlighter plot of 24 representative 3GS reads and 4 consensus sequences (con a to con d) according to the four identified sequence clusters (a-d), determined for the 2^nd^ half genome (HxB2 position 5037-9555). Mismatches compared to the master sequence con a are shown as colored bands according to the legend. The sequence reads are partitioned according to the identified clusters and separated by a gray line. Relative abundance of each viral subpopulations (cluster) as determined by 3GS is shown in brackets (%). **(B, C, D & E)** Schematic illustration of recombinant strain diversity between and within clusters a, b, c and d. For each cluster, six representative reads (upper panel) and the respective consensus sequence (middle panel, done with Recombinant drawing tool) are shown. Bootscan plots of the consensus sequences are shown in the lower panel. 3GS: Third-generation sequencing.


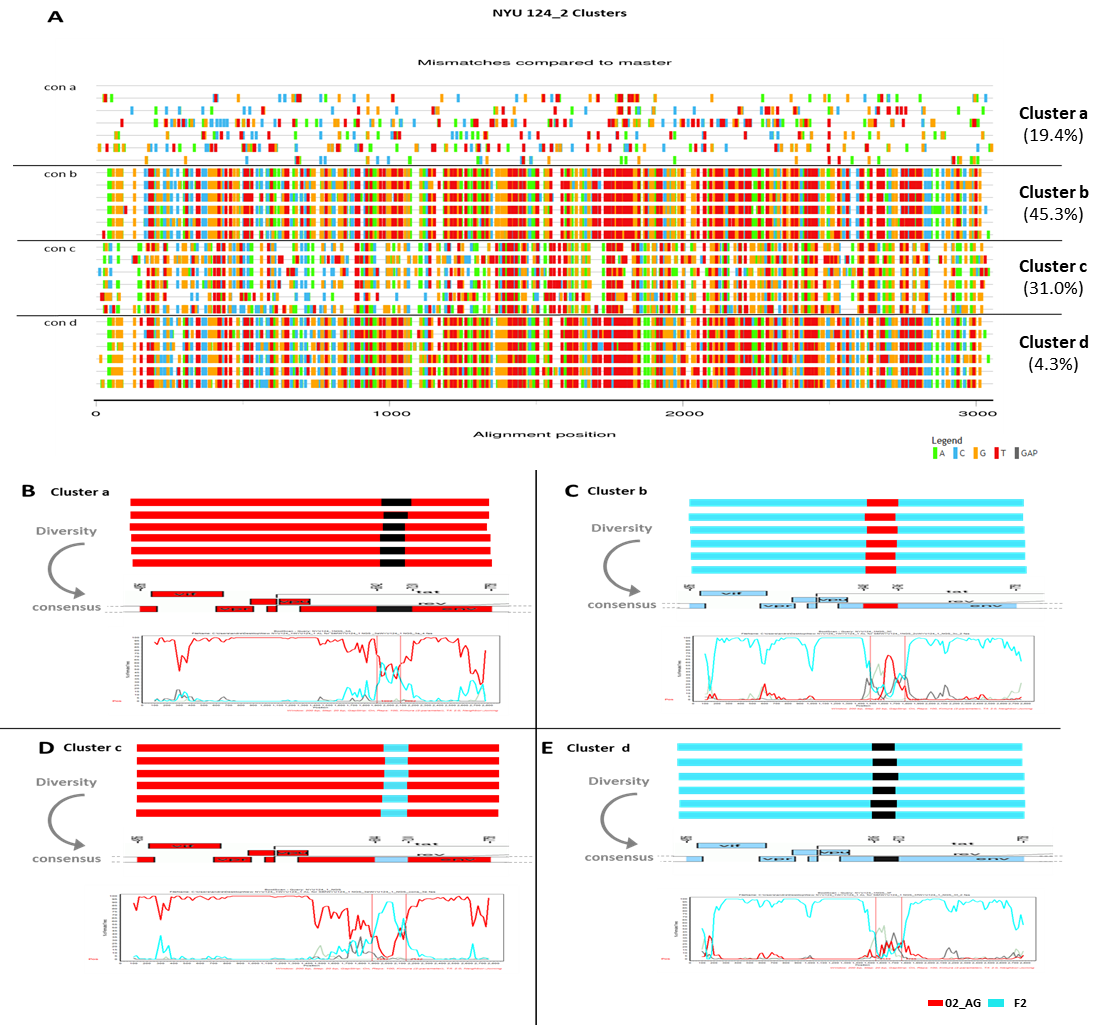


**Supplemental Figure 6. Intra-patient URF diversity in NYU124-2. (A)** Highlighter plot of 24 representative 3GS reads and 4 consensus sequences (con a to con d) according to the four identified sequence clusters (a-d), determined for the *vif/gp120* position (HxB2 position 4956-7838). Mismatches compared to con a as master sequence are shown as colored bands according to the legend. The sequence reads are partitioned according to the identified clusters and separated by a gray line. Relative abundance of each viral subpopulation (cluster) as determined by 3GS is shown in brackets (%). **(B, C, D and E)** Schematic illustration of recombinant strain diversity between and within clusters a, b, c and d. For each cluster, six representative reads (upper panel) and the respective consensus sequence (middle panel, done with Recombinant drawing tool) are shown. Bootscan plots of the consensus sequences are shown in the lower panel. 3GS: Third-generation sequencing.


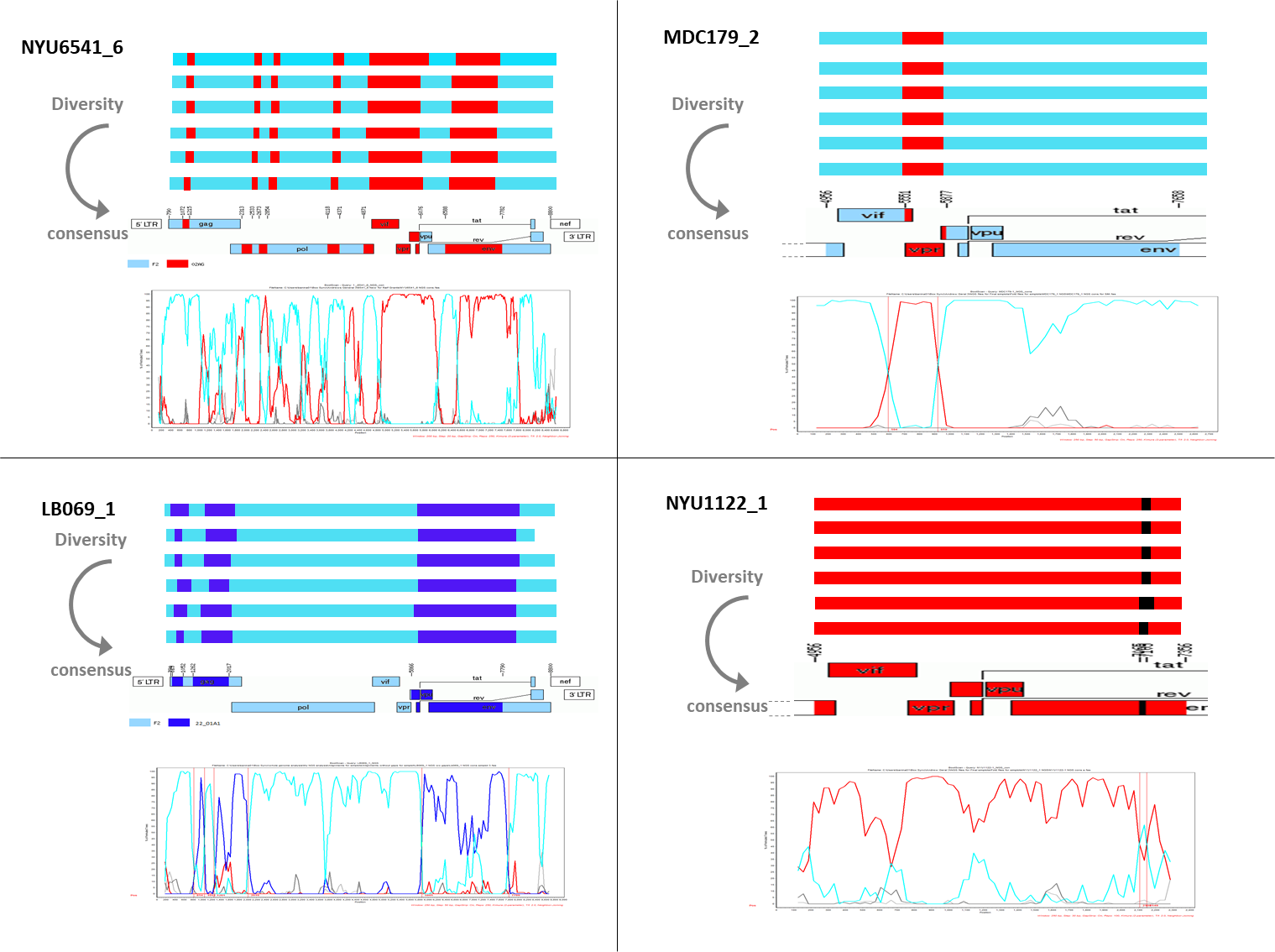


**Supplemental Figure 7. Intra-patient URF diversity in NYU6541_6, MDC179-2, LB069_1 and NYU1122_1**. Schematic illustration of recombinant strain diversity within subjects, determined for the near full genomes of NYU6541_6 and LB069_1, and for the *vif/gp120* genomic regions of MDC172-2 and NYU1122-1. Six representative 3GS reads (upper panel) and the respective consensus sequence (middle panel, done with Recombinant drawing tool) are shown per subject. 3GS: Third-generation sequencing.

N88

N156

N160

R4

K169

V172

I181mm

R4

100
 | | | | | | | | | |
B.FR.1983.HXB2-patent.JA806687_1 MRVKEKYQHL WRWGWRWGTM LLGMLMICSA TEKLWVTVYY GVPVWKEATT TLFCASDAKA YDTEVHNVWA THACVPTDPN PQEVVLVNVT ENFNMWKNDM

02_AG.CM.2002.02CM_0014BBY.AY371126_1 MRVMG-MKKN YPSLWI--TV WLSIIMMCNA -TDMWVTVYY GVPVWRDAET TLFCASDAKA YKKEVHNVWA THACVPTDPN PQEIFLKNVT EKFNMWKNNM

F2.CM.1997.CM53657.AF377956_1 MRVRE-MQRN WQHLGKWGLL FLGILIICNA ADNLWVTVYY GVPVWKEATT TLFCASDAKA YEREIHNVWA TYACVPTDPN PQELVLGNVT ENFNMWKNNM

LB016-1 MRVMG-IRKN YPLLWRWGMI IFWIMIICNA -QNLWVTVYY GVPVWREATT TLFCASDAKA FDTEVHNVWA THACVPTDPN PQEIQLKNVT ENFNMWKNDM

LB082-1 MRVRE-MQRN WQSLGKWGLL FLGILIICSA -ENLWVTVYY GVPVWRDAET TLFCASDAKA YESEVHNVWA THSCVPTDPN PQEINMENVT EEFNMWKNNM

LB069-1 MRVRA-MQRN WQHLGKWSLL FLGILIICNA ADNLWVTVYY GVPVWKEATT TLFCASDAKA YEREVHNVWA THACVPTDPN PQEVNLVNVT EHFNIWENNM

LB095-1 MRVKG-IQRN YPLLWSWGTL IFSLMIICNA -GNLWVTVYY GVPVWRDAET TLFCASDAKA YSTEAHNVWA THACVPTDPS PQEIYLENVT EEFNMWKNNM

LB104-1 MKVTG-TLMN WPPLWKGVTL ILGLVIICSA -SDLWVTVYY GVPVWRDADT TLFCASDAKA YEKEEHNVWA THACVPTDPN AREIDLLNVT ENFNVWKIIM

BDHS24-2 MRVRG-IQRN YPSFWGWGTM ILWLMITCSA -HNLWVTIYY GVPVWRDAKT ILFCASDARA YDTEAHNVWA THACVPTDPS PQEINLVNVT EQFNMWKNDM

BDHS33 MRVMG-IQRN YPILWRVGTI ICWLMIICSA -QNLWVTVYY GVPVWKEATT TLFCASDAKA YDTEAHNVWA THACVPTDPN PQEMILENVT EEFNMWKNNM

MDC131-1 MRVKG-IQRN YPLLWSWGTL IFYLMIICNA -GNLWVTVYY GVPVWRDAET TLFCASDAKA YRTEAHNVWA THACVPTDPS PQEIYLENVT EEFNMWKNNM

MDC179-2 MRVRE-MQRS WQHLGRWGLL FLGILIICNA TDNLWVTVYY GVPVWKEAKT TLFCASDAKA YEKEVHNVWA THACVPTDPD PQEL-LLNIS ENFNMWENNM

NYU119-3 MRVRG-IQRN YPSFWGWGTM ILWLMITCSA -QNLWVTVYY GVPVWRDAKT ILFCASDARA YDTEAHNVWA THACVPTDPS PQEINLVNVT EQFNMWKNDM

NYU124-2 MRVRG-IQRN YPSFWGWGTM ILWLMITCSA -QNLWVTVYY GVPVWRDAKT ILFCASDARA YDTEAHNVWA THACVPTDPS PQEINLVNVT EQFNMWKNDM

NYU129-5 MRVMG-IQRN YPLFWRWGLI IFGIMMICNA -QNLWVTVYY GVPVWKEAKT TLFCASDAKA YDREAHNVWA THACVPTDPN PQEIHLENVT EDFNMWENNM

NYU1122_1 MRVMG-IQRN CQHLLTWGIM ILGTIIFCSA VENLWVTVYY GVPVWRDADT TLFCASDAKA YETEKHNVWA THACVPTDPN PQEIHLDNVT EKFNMWKNNM

NYU1999 MRVRG-IQRN YPSFWGWGTM ILWLMITCSA -QNLWVTVYY GVPVWRDAKT ILFCASDARA YDTEAHNVWA THACVPTDPS PQEINLVNVT EQFNMWKNDM

NYU2140-1 MRVRG-IQRN YPSFWGWGTM ILWLMITCSA -QNLWVTVYY GVPVWRDAKT ILFCASDARA YDTEAHNVWA THACVPTDPS PQEINLVNVT EQFNMWKNDM

NYU6556-3 MRVRG-IQRN YPLFWRWGIL IFGLMIICNA -DKLWVTVYY GVPVWRDAET TLFCASDAKA YDTEAHNVWA THACVPTDPN PQEINLENVT EVFNMWKNNM

NYU6541-6 MRVMG-TQRN YLLLWRWGTL IFWIMMICNA -KQLWVTVYY GVPVWKEAET TLFCASDAKA YDTEAHNVWA THACVPTDPS PQEIHLENVT EPFNMWKNNM

187
 101 I----------------------V1-------------------I-----------------V2------------------
B.FR.1983.HXB2-patent.JA806687_1 VEQMHEDIIS LWDQSLKPCV KLTPLCVSLK CTDLKNDTNT NS------SS ---GRMIMEK GEIKNCSFNI STSIRGKVQK EYAFFYKLDI IPIDND----

02_AG.CM.2002.02CM_0014BBY.AY371126_1 VEQMHEDIIS LWDQSLKPCV KLTPLCVTLN CTSISNSTSN SS------IS ----IRSEMK EEIKNCSFNA TTEIRDKEKK EYALFYRLDI EQIDNS----

F2.CM.1997.CM53657.AF377956_1 VDQMHEDIIS LWDQSLKPCV QITPLCVTLN CTDVPVNITN GNST------ -LDNITLEEQ GEIKNCSFNI TTEINDIKKK ESAIFYRLDV VPINNS----

LB016-1 VEQMHEDIIS LWDQSLKPCV KLTPLCVTLQ CT-----NLT ---------- ---NVTADMK EVMTNCSFNT TTEVRDKERK TYALFYRLDV VPINKDNSGS

LB082-1 VEQMHTDVIS LWDQSLKPCV KLTPLCVTLN CQDITIESNT NTTNTTNTTN TTVTSNISVT SDMKNCSYNV TTEIRDKIKK VYSLFYRLDV EQIGN-----

LB069-1 VEQMQEDIIS LWDESLKPCV KLTPLCVTLN CTKFTKANST QN-------- ---ATDNQAQ EEIKNCSFDM TTELKDKKKR VYSLFYRLDI VELNAS----

LB095-1 VEQIHNDIIS LWDQSLKPCV KLTPLCVTLS CTDAFND--- ---------- ---------- TDTANCTYNM TTELLDKKQK VHSLFYKLDV VPMNNKTSHK

LB104-1 VEQMQEDIIS LWDESLKPCV KLTPLCVTLN CSDSSISHF- -N-------- ---DSNFNMS NDMQNCSFNM TTELRDKRKK VFSLFYRHDI VPINGD----

BDHS24-2 VDQMHTDIIS LWDQSLKPCV KLTPLCVTLK CADVNATY-- ---------- ----TNTSDS VEIRNCSFNM TTELRDKKQQ VYALFYRQDV VQINNNG---

BDHS33 VEQMHTDIIS LWEQSLKPCV SLTPLCVTLN CTDTNNTNTT NS------T- ------LGRR EEMKNCSFNV TAELRDKKQQ VYALFYKPDV VQINES----

MDC131-1 VEQMHKDIIS LWDQSLRPCV KLTPLCVTLT GNGIINNTST NS------T- ------LGRR EEMKRCAYNM TAELRDKKQK VYSIYYKPDL VKINIG----

MDC179-2 VDQMQEDIIS LWDESLKPCV KMTPLCVTLK CSDATIASAN ATNNATIKG- -NNTDILEEQ GAIKNCSFNV TTKIQDKKKL EYAFFYKLDV VEINN-----

NYU119-3 VDQMHTDIIS LWDQSLKPCV KLTPLCVTLK CADVNATY-- ---------- ----TNTSES VEIRNCSFNM TTELRDKKQQ VYALFYRQDV VQINNNG---

NYU124-2 VDQMHTDIIS LWDQSLKPCV KLTPLCVTLK CADVNATY-- ---------- ----TNTSES VEIRNCSFNM TTELRDKKQQ VYALFYRQDV VQINNNG---

NYU129-5 VEQMHKDIIS LWDQSLKPCV KLTPLCVTLQ CHNSNNNGNH ---------- ---NATIEMQ GEIKNCSFNI TTELRDKKKK ENALFYKYDV VQISRNESQ-

NYU1122-1 VEQMHTDIIS LWDQSLKPCV KLTPLCVTLQ CT-----NLT ---------- ---NVTADMK EVMTNCSFNT TTEVRDKERK TYALFYRLDV VPINKDNSGS

NYU1999 VDQMHTDIIS LWDQSLKPCV KLTPLCVTLK CADVNVTYTN TS------DV NVTYTNTSES VEIRNCSFNM TTELRDKKQQ VYALFYRQDV VQINNNG---

NYU2140-1 VDQMHTDIIS LWDQSLKPCV KLTPLCVTLK CADVNATYTN TS------ES -VEMRNTSES VEMRNCYFNM TTELRDKKQQ VYALFYRQDV VQINNNG---

NYU6556-3 VEQMHTDIIS LWDQSLKPCV QLTPLCVTLN CSNANKTIGN -S------TV NL--AADDMK KEIQNCSFNM TTELRDKKQK VYALFYRQDV EEIDEGSQGN

NYU6541-6 VEQMHIDIIS LWEQSLKPCV KLTPLCVTLN CSRPHFNDSS IS-------- HFNDS-NNMS NDMQNCSFNM TTELRDKRKK VFSLFYRLDI LPIDNNNIS

R4

N234

263
 --------------V2--------------------I | | | | | | |
B.FR.1983.HXB2-patent.JA806687_1 ---------- ---------- -----TTSYK LTSCNTSVIT QACPKVSFEP IPIHYCAPAG FAILKCNNKT FNGTGPCTNV STVQCTHGIR PVVSTQLLLN

02_AG.CM.2002.02CM_0014BBY.AY371126_1 ---------- ---------- ----NNSTYR LINCNTSTIA QACPKVTFEP IPIHYCAPAG FAILKCNDKE FNGTGPCKNV STVQCTHGIK PVVSTQLLLN

F2.CM.1997.CM53657.AF377956_1 ---------- ---------- -----TSEYR LLSCNTSTVT QACPKVSFDP IPIHYCAPAG FAILKCNDKE FNGTGLCRNV STVQCTHGIK PVVSTQLLLN

LB016-1 SS-------- ---------N NGNKTIEEYR LINCNTSVIK QACPKVTFEP IPIHYCAPAG FAILKCNERN FTGTGPCNNV STVQCTHGIK PVVSTQLLLN

LB082-1 ---------- ---------- -----SSKYI LVNCNTSTIA QACPKVTFEP IPIHYCAPAG FAILKCKDKK FNGTGPCRNV SSVQCTHGIR PVVSTQLLLN

LB069-1 ---------- ---------- -----SNEYR LINCNTSAIT QACPKVTFEP IPIHYCAPAG FAILKCNDKQ FNGTGLCKNV SSVQCTHGIK PVVSTQLLLN

LB095-1 NISS------ ---------- NESVEYREYR LINCNTSAIT QACPKVSFEP IPIHFCAPAG FAILKCREKE FNGTGPCKNV STVQCTHGIK PVVSTQLLLN

LB104-1 ---------- ---------- ----KNNQYR LINCNTSAIT QACPKVTFEP IPIHYCAPAG FAILKCKDIE FNGTGPCHNV SSVQCTHGIK PVVSTQLLLN

BDHS24-2 ---------- ---------- N---NDSEYR LINCNTSTIT QACPKVSFQP IPIHYCAPAG FAILKCNNKT FNGIGPCNNV STVQCTHGIK PVVSTQLLLN

BDHS33 ---------- ---------- ----SSNYYR LINCNASTIT QACPKVSFEP IPIHYCAPAG YAILKCNEKN FNGTGKCNNV STVQCTHGIK PVVSTQLILN

MDC131-1 ---------- ---------- ----YNEYYR LINCNTSAIT QACPKVSFEP IPIHFCAPAG FAILKCREKE FNGTGPCKNV STVQCTHGIK PVVSTQLLLN

MDC179-2 ---------- ---------- -----ESEYR LIHCNTSTIT QACPKVTFEP IPIHYCAPAG FAILKCNDKR FNGTGTCNNV STVQCTHGIK PVVTTQLLLN

NYU119-3 ---------- ---------- N---NDSEYR LINCNTSTIT QACPKVSFQP IPIHYCAPAG FAILKCNNKT FNGIGPCNNV STVQCTHGIK PVVSTQLLLN

NYU124-2 ---------- ---------- N---NDSEYR LINCNTSTIT QACPKVSFQP IPIHYCAPAG FAILKCNNKT FNGIGPCNNV STVQCTHGIK PVVSTQLLLN

NYU129-5 ---------- ---------- -------KYR LISCDTSTIT QACPKVTFEP IPIHYCAPAG FAILKCKDTK FNGKGPCKNV STVQCTHGIK PVVSTQLLLN

NYU1122-1 SS-------- ---------N NGNKTIEEYR LINCNTSVIK QACPKVTFEP IPIHYCAPAG FAILKCNERN FTGTGPCNNV STVQCTHGIK PVVSTQLLLN

NYU1999 ---------- ---------- N---NDSEYR LINCNTSTIT QACPKVSFQP IPIHYCAPAG FAILKCNNKT FNGIGPCNNV STVQCTHGIK PVVSTQLLLN

NYU2140_1 ---------- ---------- N---NDSEYR LINCNTSTIT QACPKVSFQP IPIHYCAPAG FAILKCNNKT FNGIGPCNNV STVQCTHGIK PVVSTQLLLN

NYU6556-3 GSQS------ ---------- N---GSGIYR LINCNTSTIA QACPKISFQP IPIHYCAPAG FAILKCKDKE FNGTGPCKNV STVQCTHGIK PVVSTQLLLN

NYU6541-6 ---------- ---------- ---SNSSEYR LINCNISTVT QACPKMSFEP IPIHYCAPAG FAILKCNDKN FNGTGTCKNV STVQCTHGIK PVVSTQLLLN

N301

N332/4

R4

R4

N276

264 353
 | | | I---------------------V3---------------------I | | |
B.FR.1983.HXB2-patent.JA806687_1 GSLAEEEVVI RSVNFTDNAK TIIVQLNTSV EINCTRPNNN TRKRI-RIQR GPGRAFVTI- --GKIGNMRQ AHCNISRAKW NNTLKQIASK LREQF-----

02_AG.CM.2002.02CM_0014BBY.AY371126_1 GSLAEKEIMI RSENITNNAK TIIIQLTEPV KINCTRPGNN TRKSV---RI GPGQTFYAS- -GAIIGDIRQ SHCNVSATEW NKTLQQVATQ LRKYF-----

F2.CM.1997.CM53657.AF377956_1 GSLAEGDIVI RSENISDNAK TIIVQFNRSV AINCTRPTNI TRRS---MRI GPGRVFYAT- -GTVLGDIRK AYCTINGTLW NKTLEGVAKE VQSH------

LB016-1 GSLAEERVVI RSENITNNAK SIIAQLHTPV RINCSRPSNN TRKG---VHI GPGQAFYAT- -GDITGDIRQ AHCNVSTTEW KEALGQVVTQ LSKSL-GKNE

LB082-1 GSLAEEEVMI RSENLTNNAK TIIVQLVTPV EINCTRPSNN TRKS---IHL GPGRVIYAT- -GAIMGNIRQ AHCNISKTQW SEALQLVAKQ LKQHF-----

LB069-1 GSLAEGNITI RSKNITDNAK TIIVQLAEPV TITCIRPFNN TRKS---IHI GPGRAFYAT- -GDIIGDIRQ AHCNVNKTNW NKTLGEVAQK LRQY------

LB095-1 GSLAEGDVMI RSENISDNAK TIIVQLNKPV KINCTRVGNN TRKGV---HI GPGQTFYAV- -DAVIGDIRQ AHCTVNGTEW KEAVQKVVTK LGMSF-----

LB104-1 GSLAEENIII RSENLTNNAK TIIVQLVDPV NITCIRPNNN TREGIRRIDM GPGQVFYAR- -GDITGDIRQ AYCLVNKTQW NNTLHRVAQK LQDKLKGIHT

BDHS24-2 GSLAEEKVMI RSENITNNAK TIIVQLTNPV RINCVRPNNN TRKSI---RI GPGQTFYAT- -GEVIGDIRR AHCNVSKTQW NDTLQQVATQ LKT----YFG

BDHS33 GSLSEGEIVI RSENITDNTK TIIVQLNKTV EINCTRPGNN TIKGVRRIGI GLGQTFLA-- -NSIIGDIRK AHCNVSEKEW KEALKEVANK LRNKLKGNHT

MDC131-1 GSLAEENVMI RSENISNNVK TIIVQLNKPV KINCIRPGNN TRKSIRRIDI GPGQTFYAI- -DDIIGNIRE AHCNVNGTEW NEAVRKVVTK LGIKLKGPFT

MDC179-2 GSLSEEGIMI RSKNITDNTK TIIVQFDRAI EINCTRPNNN TRKD---IQL GPGKAIYAT- -GAIIGDIKK AYCTINGTLW DETLKRVIDE FKNKT-----

NYU119-3 GSLAEEKVMI RSENITNNAK TIIVQLTNPV RINCTRPNNN TRKSI---RI GPGQTFYAT- -GEVIGDIRR AHCNVSKTQW NDTLQQVATQ LKT----YFG

NYU124-2 GSLAEEKVMI RSENITNNAK TIIVQLTNPV RINCTRPNNN TRKSI---RI GPGQTFYAT- -GEVIGDIRR AHCNVSKTQW NDTLQQVATQ LKT----YFG

NYU129-5 GSLAEEEIAI RSENITNNVK TIIVQLKNPV NITCIRPGNN TRKSMRRIGI GPGQTFYAT- -GDIIGDIRQ AHCNVSGTKW HGTLHQVVTQ LRNKLKGTST

NYU1122_1 GSLAEERVVI RSENITNNAK IIIVQLVQPV TIKCIRPNNN TRKS---IRI GPGQAFYAT- -GDIIGDIRQ AHCNVTRSRW NKTLQEVAEK LRTY------

NYU1999 GSLAEEKVMI RSENITNNAK TIIVQLTNPV RINCIRPNNN TRKSI---RI GPGQTFYAT- -GEVIGDIRR AHCNVSKTQW NNTLQQVATQ LKT----YFG

NYU2140_1 GSLAEEKVMI RSENITNNAK TIIVQLTNPV RINCTRPNNN TRKSI---RI GPGQTFYAT- -GEVIGDIRR AHCNVSKTQW NDTLQQVATQ LKT----YFG

NYU6556-3 GSLAEEKIMI RSENITNNAK TIIVQFTTPV RINCIRPNNN TRTSM---RI GPGQAFYAT- -GDIIGDIRA AHCNVSKTEW NTTLQQVAKQ LGM------Y

NYU6541-6 GSLAEEDIII RSENITDNIK NIIVQLNRSI EINCTRPGNN TRKSV---RI GPGQTFYAT- -GDIIGDIRQ AHCNISATKW NSTLQQVAAQ LRRHF-----

R4

R4

R4

R4

354 450
 | | | I----------------V4------------------I | | | |
B.FR.1983.HXB2-patent.JA806687_1 GNNKTIIFKQ SSGGDPEIVT HSFNCGGEFF YCNSTQLFNS TWFNST-WST EGSNNTEGSD TITLPCRIKQ IINMWQKVGK AMYAPPISGQ IRCSSNITGL

02_AG.CM.2002.02CM_0014BBY.AY371126_1 KNDTTIKFAS SSGGDVKITT HSVNCGGEFF YCNTSNLFNS TWISNSTWNS -S--SSTGSD IITLQCRIKQ IVNMWQKVGQ AMYAPPIRGE IRCNSNITGL

F2.CM.1997.CM53657.AF377956_1 -LNKSITFAP SSGGDLEVTT HSFNCRGEFF YCNTVALFNA TN-------- MTNAMNRSNG IITLPCRIRQ IVNMWQRVGR AMYAAPIAGQ IQCNSSITGL

LB016-1 TKNTTIIFAN SSGGDVEITT HSFNCGGEFF YCNTSNLFNS TWGSNTTTSW NTSSIG-TNE TITIQCRIKQ IVRMWQRVGQ AMYAPPIPGK IRCNSSITGL

LB082-1 -NNATIKFNE SSGGDYEIQT HSFNCGGEFF YCNTSSLFDS A--WLNNETG IGQNITESNN TITLQCRIKQ FINMWQRVGQ ALYAPPIPGN ITCRSNITGL

LB069-1 -WNKTIIFTS SSGGDIELTT HTFNCRGEFF YCNTSNLFNW NGTWNGNTT- -------GND TITLQCRIKQ IINMWQRVGQ AMYAPPIPGE IKCESNITGL

LB095-1 QNATKIIFAN ASGGDIEITT HTFNCGGEFF YCNTTKLFNS IWSKNSGNWS -S--TNGTND TITLQCRIKQ IVRMWQRVGQ AMYAPPIRGN ITCGSNITGL

LB104-1 TGNTTIIFTN SSGGDLEITT HSFNCKGEFF YCNTTNLFNS I--HSDSMTN ------TGDG NIILQCRIKQ IINMRQRAGK AIYAPPIRGR IQCESNITGI

BDHS24-2 GNTTTIIFNK SSGGDLEITT HSFNCGGEFF YCNTTDLFS- -WNSSSPWKN IT-----SND SITLQCRIKQ IVRMWQQVGQ AMYAPPIPGI IRCESNITGI

BDHS33 TGNKTIIFAK ASGGDLEITT HSFNCGGEFF YCNTSQLFN- -----STWNS ST--PSTGNR TITLQCKIKQ IVRMWQKVGQ AMYAPPISGN IRCNSNITGL

MDC131-1 TRNAKIIFAN ASGGDIEITT HTFNCGGEFF YCNTTRLFNS IWS------N IT--NSTAND TITLQCRIKQ IVRMWQRVGQ AMYAPPIQGE IKCESNITGL

MDC179-2 NSNNTVTFGS PTGGDLEIMM HTLNCGGEFF YCNTTVLFNT TG-------- -------LKD SITIPCKIKQ IINMWQRVGR AMYAPPIAGK IQCNSSITGL

NYU119-3 GNTTTIIFNK SSGGDLEITT HSFNCGGEFF YCNTTDLFS- -WNSSSPWKN IT-----SND SITLQCRIKQ IVRMWQQVGQ AMYAPPIPGI IRCESNITGI

NYU124-2 GNTTTIIFNK SSGGDLEITT HSFNCGGEFF YCNTTDLFS- -WNSSSPWKN IT-----SND SITLQCRIKQ IVRMWQQVGQ AMYAPPIPGI IRCESNITGI

NYU129-5 TNNTTIIFDK PSGGDLEITT HSFNCGGEFF YCNTSNLFNS TWGNNT---- ----CSSSNI NITLQCRIKQ IVNMWQTVGQ AMYAPPIQGE IRCESNITGL

NYU1122-1 FGNKTIIFAN SSGGDLEITT HSFNCGGEFF YCNTSGLFNS TWYVNSTWN- DTDSTQESND TITLPCRIKQ IINMWQRAGQ AMYAPPIPGV IKCESNITGL

NYU1999 GNTTTIIFNK SSGGDLEITT HSFNCGGEFF YCNTTDLFS- -WNSSNPWKN IT-----SND SITLQCRIKQ IVRMWQQVGQ AMYAPPIPGI IRCESNITGI

NYU2140-1 GNTTTIIFNK SSGGDLEITT HSFNCGGEFF YCNTTDLFS- -WNSSSPWKN IT-----SND SITLQCRIKQ IVRMWQQVGQ AMYAPPIPGI IRCESNITGI

NYU6556-3 FGNKTIIFAN HSGGDVEITT HSFNCGGEFF YCNTSGLFNS TWHNSTWDSN ET--ASATND TITLRCRIKQ FVNMWQKVGR AMYAPPIPGV IRCESNITGL

NYU6541-6 -SNKTIIFKS SSGGDIEITT HSFNCAGEFF YCNTTPLFNS TWKSNDTASS N------PND TITLQCRIKQ IVNMWQKVGQ AMYAPPISGN IMCNSTITGI

R4

R4

R4

R4

R4

451 542
 | | I--V5---I| | | gp120 ▼ gp41 | |
B.FR.1983.HXB2-patent.JA806687_1 LLTRDGGNSN -----NESEI FRPGGGDMRD NWRSELYKYK VVKIEPLGVA PTKAKRRVVQ --REKRA-VG IGALFLGFLG AAGSTMGAAS MTLTVQARQL

02_AG.CM.2002.02CM_0014BBY.AY371126_1 LLTRDGGDRG NSGGANGTET FRPGRENMKD NWRSELYKYK VVKIEPLGVA PSHAKRRVVE --REKRA-VG MGAVFLGFLG AAGSTMGAAS ITLTVQARQL

F2.CM.1997.CM53657.AF377956_1 ILTRDGGKNN TNN-----DT LRPGGGDMRD NWRSELYKYK VVKIEPLGVA PTKAKRQVVK REREKRA-VG IGAVLLGFLG AAGSTMGAAS MTLTVQARQL

LB016-1 LLTRDGGNNS ----S-DNET FRPGGGDMRD NWRSELYKYK VVKIEPLGVA PTHARRRVVE --REKRA-VG LGAVFLGVLG AAGSTMGAAS ITLTVQARQL

LB082-1 LLTRDGGNNT SDNGT-VIET FRPGGGDMRD NWRSELYKYK VVKLEPLGIA PTEARRRVVE --REKRA-IG LGAFFLGFLG AAGSTMGAAS LTLTVQARQL

LB069-1 LLTRDGGNST D-N-----ET FRPGGGDMRD NWRSELYKYK VVKIEPLGVA PNKARRRVVG --REKRA-VG LGAVFLGFLS AAGSTMGAAS LTLTVQARQL

LB095-1 LLTRDGDGGA ----HSPNET FRPGGGNMKD NWRSELYKYK VVQIKPLGVA PTQAKRRVVE --REKRA-VG LGAVFLGFLG AAGSTMGAAS LTLTVQARQL

LB104-1 LLTRDGGNTS NSN-----ET FRPGGGDMRD NWRSELYKYK VVKIEPLGVA PTKARRRVVE --REKRA-FG LGAMFLGFLG TAGSTMGAAS LTLTVQARQL

BDHS24-2 LLTRDGGNNN ----TNNTEV FRPGGGDMRD NWRSELYKYK VVKIEPLGVA PSRARRRVVE --REKRA-VG LGAFFLGFLG AAGSTMGAAS ITLTIQARQL

BDHS33 LLTRDGVGNN ----S-TNET FRPGGGDMRN NWRSELYKYK VVQIKPLGVA PTQAKRRVVQ --REKRA-IG LGAVFIGFLG AAGSTMGAAS ITLTVQARQL

MDC131-1 LLTRDGGHNN -----GDNET FRPGGGNMKD NWRSELYKYK VVQIKPLGVA PTHAKRRVVE --REKRA-VG LGAVFLGFLG AAGSTMGAAS ITLTVQARQL

MDC179-2 LLTRDGVNDT KN------ET LRPGGGDMRD NWRSELYKYK VVQIEPLGVA PTRAQRRVVQ REK-RAV-VG LGAMFIGFLG AAGSTMGAAS ITLTVQARQL

NYU119-3 LLTRDGGNNN ----TNNTEV FRPGGGDMRD NWRSELYKYK VVKIEPLGVA PSRARRRVVE --REKRA-VG LGAFFLGFLG AAGSTMGAAS ITLTIQARQL

NYU124-2 LLTRDGGNNN ----TNNTEV FRPGGGDMRD NWRSELYKYK VVKIEPLGVA PSRARRRVVE --REKRA-VG LGAFFLGFLG AAGSTMGAAS ITLTVQARQL

NYU129-5 LLTRDGGGKT ----N-NTEI FRPGGGDMRD NWRSELYKYK VVKIEPLGVA PTHAKRRVVE --REKRA-VG LGAVFLGFLG AAGSTMGAAS ITLTVQARQL

NYU1122-1 LLTRDGGKDN ----N-VNET FRPGGGDMRD NWRSELYKYK VVEIEPLGVA PTRAKRRVVE --REKRA-VG IGAVFLGFLG AAGSTMGAAS ITLTVQARQL

NYU1999 LLTRDGGNNN ----TNNTEV FRPGGGDMRD NWRSELYKYK VVKIEPLGVA PSRARRRVVE --REKRA-VG LGAFFLGFLG AAGSTMGAAS ITLTVQARQL

NYU2140-1 LLTRDGGNNN ----TNNTEV FRPGGGDMRD NWRSELYKYK VVKIEPLGVA PSRARRRVVE --REKRA-VG LGAFFLGFLG AAGSTMGAAS ITLTVQARQL

NYU6556-3 LLTRDGGNNN ----NSVNET FRPGGGDMRD NWRSELYKYK VVQIEPLGVA PTHARRRVVQ --REKRA-VG LGAVFLGFLG AAGSTMGAAS ITLTVQARQL

NYU6541-6 LLTRDGGINN ----TEEEET FRPGGGDMRD NWRSELYKYK VVKIEPLGVA PTYAKRRVVE --REKRA-VG LGAVFLGFLG AAGSTMGAAS ITLTVQARQL

N637

N611

543 641
 | | | | | | | | | |
B.FR.1983.HXB2-patent.JA806687_1 LSGIVQQQNN LLR-AIEAQQ HLLQLTVWGI KQLQARILAV ERYLKDQQLL GIWGCSGKLI CTTAVPWNAS WSN-KSLEQI WNHTTWMEWD REINNYTSLI

02_AG.CM.2002.02CM_0014BBY.AY371126_1 LSGIVQQQNN LLR-AIEAQQ HLLKLTVWGI KQLQARVLAL ERYLRDQQLL GIWGCSGKLI YTTTVPWNSS WSN-KTFNDI WDNMTWLQWD KEISNYTEAI

F2.CM.1997.CM53657.AF377956_1 LSGIVQQQNN LLK-AIEAQQ HLLQLTVWGI KQLQARILAV ERYLKDQQLL GIWGCSGKLI CTTNVPWNSS WSN-KSQNEI WENMTWMQWE KEISNYTGTI

LB016-1 LSGIVQQQSN LLR-AIEAQQ HMLRLTVWGI KQLQARVLAL ERCLKDQQLL GIWGCSGKLI CTTTVPWNSS WSN-KTYRDI WDSMTWLQWD KEISNYTSIM

LB082-1 LSGIVQQQNN LLK-AIEAQQ LMLKLTVWGI KQLQARVLAV ERYLKDQQLR GIWGCSGKLI CTTNVPWNSS WSN-KSQSDI WHNMTWLEWD REINKYTTTI

LB069-1 LSGIVQQQNN LLR-AIEAQQ HMLRLTVWGI KQLQARVLAV ERYLKDQQLL GIWGCSGKLI CPTSVPWNAS WSN-KSQDEI WGNMTWLQWD KEISNYTDII

LB095-1 LSGIVQQQSN LLR-AIEAQQ HLLKLTVWGI KQLQARVLAL ERYTRDQQLL GIWGCSGKLI CTTSVPWNST WSN-KSYNDI WANMTWLQWD KEINNYTEII

LB104-1 LSGIVQQQSN LLR-AIEAQQ NMLRLTVWGI KQLQARVLAV ERYLKDQQLL GIWGCSGKLI CTTNVPWNTS WSN-KTQDQI WENMTWLEWE REISNYTDII

BDHS24-2 LSGIVQQQSN LLR-AIEAQQ HLLKLTVWGI KQLQARVLAL ERYPRDQQLL GIWGCSGKLI CTTSVPWNST WSN-KSYNDI WDNMTWLQWD KEINNYTEII

BDHS33 LSGIVQQQSN LLR-AIEAQQ HLLKLTVWGI KQLQARVLAL ERYTRDQQLL GIWGCSGKLI CTTSVPWNST WSN-KSYNDI WDNMTWLQWD KEINNYTEII

MDC131-1 LSGIVQQQSN LLR-AIEAQQ HLLKLTVWGI KQLQARVLAL ERYLQDQQLL GIWGCSGKLI CTTTVPWNSS WSN-KNLSAI WDNMTWQRWD REISNYTETI

MDC179-2 LYGIVQQQSN LLR-AIEAQQ HLLQLTVWGI KQLQARILAV ERYLKDQQLL GLWGCSGKLI CTTTVPWNSS WSN-RSYGEI WENLTWIQWE KEISNYTSTI

NYU119-3 LSGIVQQQSN SADGLIEAQQ HLLKLTVWGI KQLQARVLAL ERYTRDQQLL GIWGCSGKLI CTTSVPWNST WSN-KSYNDI WDNMTWLQWD KEITNYPEII

NYU124-2 LSGIVQQQSN LLR-AIEAQQ HLLKLAVWGI KLLQARVLAL ERFARDQQVL GIWGCSGKLI CTTSVPWNST WSN-KSYNDI WDNMTWLQWD KEINNYTEII

NYU129-5 LSGIVQQQSN LLK-AIEAQQ QMLRLTVWGI KQLQARVLAL ERYLKDQQLL GIWGCSGKLI CTTAVAWNST WSN-KTYKEI WDNMTWMRWD REINNYTEII

NYU1122-1 LSGIVQQQNN LLR-AIEAQQ HLLKLTVWGI KQLQARVLAV ERYLRDQQLL GIWGCSGKLI CTTNVPWNSS WSN-KSLDEI WNNMTWLQWD KEINNYTQLI

NYU1999 LSGIVQQQSN LLR-AIEAQQ HLLKLTVWGI KQLQARVLAL ERYTRDQQLL GIWGCSGKLI CTTSVPWNST WSN-KSYNDI WDNMTWLQWD KEINNYTEII

NYU2140-1 LSGIVQQQNN LLK-AIEAQQ HLLQLTVWGI KQLQARLLAV ERYLKDQQLL GIWGCSGKLI CTTNVPWNST WSN-KSQDEI WEKMTWMQWE KEISNHTGEI

NYU6556-3 LSGIVQQQSN LLR-AIEAQQ HLLKLTVWGI KQLQARVLAL ERYLRDQQLL GIWGCSGKLI CTTTVPWNST WSN-KTYSDI WDNMTWLQWD KEISSYTDVI

NYU6541-6 LSGIVQQQSN LLK-AIEAQQ HLLKLTVWGI KQLQARVLAL ERYLRDQQLL GIWGCSGKLI CTTTVPWNSS WSN-KTYDYI WDNMTWQQWD EEISNYTETI

642 738
 | | | | | | | | | |
B.FR.1983.HXB2-patent.JA806687_1 HSLIEESQNQ QEKNEQELLE LDKWASLWNW FNITNWLWYI KLFIMIVGGL VGLRIVFAVL SIVN---RVR QGYSPLSFQT HLPTPRG-PD RPEGIEEEGG

02_AG.CM.2002.02CM_0014BBY.AY371126_1 YNLIEEAQNQ QEKNEQDLLA LDKWKSLWNW FNITNWLWYI RIFIIIIGSL IGLRIVFAVL AIIN---RVR QGYSPLSFQI LTHHQRE-PD RPGRIEEGGG

F2.CM.1997.CM53657.AF377956_1 YKLIENAQNQ QEKNEQDLLA LDKWDNLWSW FTITNWLWYI KLFIMIVGGL IGLRIVFAVL AVIN---RVR QGYSPLSLQT LTPSRRE-PE RPGGIEEEGG

LB016-1 YNLIEESQSQ QEKNEQELLE LDKWSSLWNW FDITKWLWYI KIFIMIVGGL IGLRIVFTVL NIIN---RVR QGYSPLSFQT LTHHQRE-PD RPRRIEEEGG

LB082-1 YDLLEESQMQ QEKNEKDLLS LDKWASLWNW FNISNWLWYI KIFIMIVGGL IGLRIVFAVL SIIN---RVR QGYSPVSLQT LIPNPRG-PD RPGEIEEEGG

LB069-1 YNLIEESQNQ QEKNEQDLLA LDKWSTLWTW FDISNWLWYI RIFIMIVGGL IGLRIVFAVI SIIN---SVR QGYSPLSLQT LLPSPRG-PD RPGEIEEGGG

LB095-1 YQLIEESQNQ QEKNEQDLLA LDNWASLWNW FSITNWLWYI KIFIIIIGSL IGLRIVCAVL NIINRVNRVR QGYSPLSFQT LTHHQRE-PD RPERIEEGGG

LB104-1 YGLLEESQIQ QEKNEQDLLA LDKWSSLWNW FDISRWLWYI KIFIMIVGGL IGLRIVFTVL SVIN---RVR QGYSPLSLQT LLPTLRE-PD RPGEIEEEGG

BDHS24-2 YQLIEESQNQ QEKNEQDLLA LDNWASLWNW FSITNWLWYI KIFIIIIGSL IGLRIVCAVL NIIN---RVR QGYSPLSFQT LTHHQRE-PD RPERIEEGGG

BDHS33 YQLIEESQNQ QEKNEQDLLA LDNWASLWNW FSITNWLWYI KIFIIIIGSL IGLRIVCAVL NIIN---RVR QGYSPLSFQT LTHHQRE-PD RPERIEEGGG

MDC131-1 YNLIEQSQNQ QEKNEQDLLA LDKWASLWTW FDITNWLWYI KIFIMIVGGL IGLRIVFTVL NIIN---RVR QGYSPLSFQT LTHHQRE-AD RPGRIEEGDG

MDC179-2 YSLIAEAQSQ QEENEFKLLE LDKWDSLWNW FSISNWLWYI KIFIMIVGGL IGLRIVFTVL SVVR---RVR QGYSPLSLQT LIPSSRG-PD RPGRIEEEGG

NYU119-3 YHLIEQSQNQ PEKNEQDLLA LDIGQVSRIG SSITNWLWYI KIFIIIIGSL IGLRIVCAVL NIIN---RVR QGYSPLSFQT LTHHQRE-PD RPERIEEGGG

NYU124-2 YQLIEESQNQ QEKNEQDLLA LDNWASLWNW FSITNWLWYI KIFIIIIGSL IGLRIVCAVL NIIN---RVR QGYSPLSFQT LTHHQRE-PD RPERIEEGGG

NYU129-5 YELLEQSQNQ QERNEQDLLA LDKWASLWTW FDVTYWLWYI KMFIMIVGGL IGLRIVFAVL NTLK---RVR QGYSPLSFQT LTHHQRG-PD RPEKTEEGGG

NYU1122-1 YRLIEESQNQ QEKNEKELLE LDKWANLWSW FDISNWLWYI KIFIIIVGGL IGLRIVFAVL SVIN---RVR QGYSPLSFQT HTPNPRG-LD RPERIEEEDG

NYU1999 YQLIEESQNQ QEKNEQDLLA LDNWASLWNW FSITNWLWYI KIFIIIIGSL IGLRIVCAVL NIIN---RVR QGYSPLSFQT LTHHQRE-PD RPERIEEGGG

NYU2140-1 YSLIEKAQNQ QESNEKELLA LDKWNDLWNW FDITSWLWYI KIFIMIVGGL IGLRIVFAVL SVVN---RVR QGYSPLSFQT LTHHQRE-PD RPERIEEGGG

NYU6556-3 YRLIEESQNQ QEKNEQDLLA LDKWASLWSW FDITNWLWYI RIFIMIVGGL IGLRIVFAVL NVIN---RVR QGYSPLSFQI PNHHQRE-PD RPERIEEGGG

NYU6541-6 YRLIEESQNQ QEKNEKDLLA LDKWADLWSW FSITKWLWYI RIFIMIVGGL IGLRIVFAVL NIIN---RVR QGYSPLSFQT LTHHQREPPD RPGRIEEGDG

739 832
 | | | | | | | | | |
B.FR.1983.HXB2-patent.JA806687_1 ERDRDRSIRL VNGSLALIWD DLRSLCLFSY HRLRDLLLIV TRIVELLGR- ------RGWE ALKYWWNLLQ YWSQELKNSA VSLLNATAIA VAEGTDRVIE

02_AG.CM.2002.02CM_0014BBY.AY371126_1 EQDKDRSVRL VSGFLKLIWD DLRSLFLFSY HRLRDFVLIA TRTVELLGHS SLRGLRLGWE ALKHLWNLLL YWGQELKNSA TNLLDTVAIA VANWTDRVIE

F2.CM.1997.CM53657.AF377956_1 EQDKTRSVRL VSGFLALAWD DLRSLCLFSY RHLRDFILIA ARTVN----- --KGLIRGWE ILKYLGNLAQ YWGREIKNSA IDLLNTTAIV VAEGTDRIIE

LB016-1 EQDRDRSERL VSGFLALAWD DLRSLCLFSY HRLRDLVLIA ARGVELLGRN SLKGLRLGWE ALKLLGNFLL YWGRELKNSA INLLDTLAIA VANWTDRVIE

LB082-1 EQGKGRSVRL VSGFLALAWD DLRSLCLFSY RLLRDFILTA ARTVD----- --RGLKGGWE VLKYLWNLTR YWSQELKNSA ISLLNTTAIV VAEGTDRIIE

LB069-1 EQDRDRSVRL ASGFLTLVWN DLRSLFLFIY HLLRDFILIA ARTVD----- --RGLKGGWN VLKYLWNLAQ YWGQELKNSA TSLLDTTAIV VAEGTDRILE

LB095-1 EQDRDRSVRL VNGFFALIWD DLRNLCLFSY HRLRDFVWIV ARGVELLGHS SLKGLRLGWE ALKFLGNLLS YWGRELKNSA INLLDTIAIA TGNWTDRVIE

LB104-1 EQDKDRSVRL VSGFLALAWD DLRSLCLFSY RHLRDFILLA TRTVKLLGHS SLRGLIAGWE ILKYLWSLVQ YWSQELKNRA VSLLNTTAVV VAEGTDRIIE

BDHS24-2 EQDRDRSVRL VNGFFALIWD DLRNLCLFSY HRLRDFVWIV ARGVELLGHS SLKGLRLGWE ALKFLGNLLS YWGRELKNSA INLLDTIAIA TGNWTDRVIE

BDHS33 EQDRDRSVRL VNGFFALIWD DLRNLCLFSY HRLRDFVWIV ARGVELLGHS SLKGLRLGWE ALKFLGNLLS YWGRELKNSA INLLDTIAIA TGNWTDRVIE

MDC131-1 EQDRGGSVRL VDGFLALAWD DLRSLCLFSY HRLRDLVLIA ARGVELLGHS VLKGLRLGWE ALKFLGNLLS YWGRELKNSA INLLDTVAIA TANGTDRVIE

MDC179-2 EQDKDRSVRL VSGFLALAWD DLRNLCLFSY RHLRDFILIA ARTVN----- --RGLQLGWE ALKYLWNLAQ YWGQELKNSA ISLLNTTAVV VAEGTDRILE

NYU119-3 EQDRDRSVRL VNGFFALIWD DLRNLCLFSY HRLRDFVWIV ARGVELLGHS TLKGLRLGWE ALQFLGNLLS YWGRELPNTA INLLDTIAIA TGNWTDRVIE

NYU124-2 EQDRDRSVRL VNGFFALIWD DLRNLCLFSY HRLRDFVWIV ARGVELLGHS SLKGLRLGWE ALKFLGNLLS YWGRELKNSA INLLDTIAIA TGNWTDRVIE

NYU129-5 EQDRDRSVRL VSGFLALAWD DLRNLCLFSY HRLKDFLLIV ARGVELLGRS SLKGLRLGWE ALKYLGNLLS YWGQELKNSA INLLDTIAIA VANWTDRVIE

NYU1122-1 EQGRGRSIRL VSGFLALAWD DLRSLCLFSY HRLRDFILIA ARTVELLGHS SLKGLRLGWE GIKYLWNLLS YWGRELKISA INLVDTIAIA VAGWTDRVIE

NYU1999 EQDRDRSVRL VNGFFALIWD DLRNLCLFSY HRLRDFVWIV ARGVELLGHS SLKGLRLGWE ALKFLGNLLS YWGRELKNSA INLLDTIAIA TGNWTDRVIE

NYU2140-1 EQDRDRSVRL VNGFFALIWD DLRNLCLFSY HRLRDFVWIV ARGVELLGHS SLKGLRLGWE ALKFLGNLLS YWGRELKNSA INLLDTIAIA TGNWTDRVIE

NYU6556-3 EQDRDRSVRL VNGFFALIWD DLRSLCLFLY HRLRDLVLIA ARSVELLGHS SLKGLRLGWE ALKLLGNLIL YWGRELKNSA INLLDTIAIA VANWTDRVIE

NYU6541-6 EQDKNGSVRL VSGFLALVWD DLRSLCLFSY RHLRDFILIA ARIVN----- --RGLTGGWE ALKYLWNLVL YWGQELKNSA ISLLNTTAIV VAEGTDRVID

833 855
 | |
B.FR.1983.HXB2-patent.JA806687_1 VVQGACRAIR HIPRRIRQGL ERILL*

02_AG.CM.2002.02CM_0014BBY.AY371126_1 IGRRFGRAIL NIPTRIRQGF ERALL*

**Sites of immune pressure in RV144**

**N-Glycosylation sites of bnAbs**

**Deviation/Resistance**

**Sites of resistance to CD4bs bnAbs**

F2.CM.1997.CM53657.AF377956_1 VLQRAGRAIL HIPRRIRQGA ERALL*

LB016-1 IGQRIGNAIR NIPRRIRQGA ERFLL*

LB082-1 VLQRAGRAVL HIPRRIRQGF ERALL*

LB069-1 FLQRAGRAVL HIPRRIRQGF ERALL*

LB095-1 IGQRFVRAIL NIPRRIRQGA ERALN*

LB104-1 VLQTAGRAFL NIPRRIRQGL ERALL*

BDHS24-2 IGQRFVRAIL NIPRRIRQGA ERALN*

BDHS33 IGQRFVRAIL NIPRRIRQGA ERALN*

MDC131-1 IIQRAGRAII NIPTRIRQGL ERALL*

MDC179-2 VLQRAGRAVL HVPRRIRQGF ERALL*

NYU119-3 IGQRFVRAIL NIPRRIRQGA ERALN*

NYU124-2 IGQRFVRAIL NIPRRIRQGA ERALN*

NYU129-5 IGQRVGRAII NIPTRIRQGF ERALL*

NYU1122-1 IAQRIGRAIL HIPVRIRQGL ERALL*

NYU1999 IGQRFVRAIL NIPRRIRQGA ERALN*

NYU2140-1 IGQRFVRAIL NIPRRIRQGA ERALN*

NYU6556-3 VGQRAGRALL HIPRRIRQGC ERALL*

NYU6541-6 ALQRAGRAVL HIPRRIRQGF ERILL*

**Supplemental Figure 8. Env Amino acid alignment with indicated N-glycosylation sites, bnAb epitopes and sites of immune pressure.** Amino acid alignment (Clustal Omega) of functional Env sequences from 17 bulk-amplified URFs (LB089-1 not included because of a frame shift in Env) in comparison to subtype B (HxB2), CRF02_AG (0014BBY) and F2 (CM53657) reference sequences. Numbering of amino acid residues is based on HxB2 Env. N-glycosylation sites are highlighted in red (N-glycosite tool of the LANL database). N-glycosylation sites critical for selected bnAbs are boxed in red with yellow background: N88 (gp120/gp41 interphase bnAb 35O22), N156 and N160 (V2 glycan bnAbs, *e.g* PG9/PG16), N234 and N276 (gp120/gp41 interphase bnAb 8ANC195), N301 and N332/334 (V3 glycan bnAbs, *e.g.* PGT121/PGT128) as well as N611 and N637 (gp120/gp41 interphase bnAb PGT151). Sites of immune pressure in the RV144 vaccine trial [K169, V172, or mismatch (mm) at I181] and sites of resistance to CD4bs bnAbs are boxed in green and blue, respectively, according to deCamp *et al*., 2014 [3], Rolland et al., 2012 [4], and Courtney et al., 2017 [5]. Deviant/resistance conferring residues are highlighted with a red box.

**Supplemental Table 1. Comparison of phylogenetic and Simplot-based subtype classifications of recombinant fragments.** The table summarizes the results of the RAxML-based phylogenetic subtype classification for recombinant fragments >900 bp in comparison with Simplot analyses. Recombinant fragments are numbered according to their appearance in the bulk NFGS from 5’ to 3’ and as shown in **Figures 1 and S2**. The HIV-1 positions (according to HxB2) and lengths of the fragments are provided in base pairs (bp).

| **Subject ID** | **recombinant fragment #** | **subtype classification** | | **HIV-1 position according to HxB2 (bp)** | | **length (bp)** |
| --- | --- | --- | --- | --- | --- | --- |
|  |  | **Simplot** | **RAxML tree** | **start** | **end** |  |
| **LB016_1** | **3** | **02_AG** | **02_AG** | **1234** | **3535** | **2301** |
|  | **10** | **02_AG** | **02_AG** | **5295** | **6672** | **1377** |
|  | **12** | **02_AG** | **02_AG** | **7152** | **8120** | **968** |
| **NYU129-5** | **3** | **02_AG** | **02_AG** | **1529** | **3823** | **2294** |
|  | **5** | **02_AG** | **02_AG** | **4089** | **5140** | **1051** |
|  | **7** | **02_AG** | **02_AG** | **5236** | **6790** | **1554** |
|  | **9** | **02_AG** | **02_AG** | **6865** | **8821** | **1956** |
| **BDHS 33** | **1** | **02_AG** | **02_AG** | **890** | **2611** | **1721** |
|  | **2** | **F2** | **F2** | **2612** | **5061** | **2449** |
|  | **3** | **02_AG** | **02_AG** | **5062** | **6666** | **1604** |
|  | **7** | **02_AG** | **02_AG** | **7678** | **8749** | **1071** |
| **LB095_1** | **3** | **02_AG** | **02_AG** | **1552** | **2531** | **979** |
|  | **9** | **02_AG** | **02_AG** | **4084** | **6671** | **2587** |
|  | **13** | **02_AG** | **02_AG** | **7663** | **9489** | **1826** |
| **LB069-1** | **4** | **22_01A1** | **22_01A1** | **1790** | **2691** | **901** |
|  | **5** | **F2** | **F2** | **2692** | **3851** | **1159** |
|  | **9** | **F2** | **F2** | **4473** | **6431** | **1958** |
|  | **10** | **22_01A1** | **22_01A1** | **6432** | **8355** | **1923** |
|  | **11** | **F2** | **F2** | **8356** | **9286** | **930** |
| **LB082-1** | **4** | **A1** | **A1** | **2577** | **4064** | **1487** |
|  | **14** | **A1** | **A1** | **6815** | **8371** | **1556** |
| **NYU6556_3** | **1** | **02_AG** | **02_AG** | **784** | **2543** | **1759** |
|  | **3** | **02_AG** | **02_AG** | **2748** | **4169** | **1421** |
|  | **7** | **02_AG** | **02_AG** | **4835** | **9116** | **4281** |
| **NYU1999_1** | **1** | **02_AG** | **02_AG** | **785** | **5112** | **4327** |
|  | **3** | **02_AG** | **02_AG** | **5324** | **9305** | **3981** |
| **NYU2140_1** | **1** | **02_AG** | **02_AG** | **785** | **7754** | **6969** |
|  | **3** | **02_AG** | **02_AG** | **8348** | **9294** | **946** |
| **NYU1122_1** | **1** | **02_AG** | **02_AG** | **798** | **2746** | **1948** |
|  | **3** | **02_AG** | **02_AG** | **2940** | **4453** | **1513** |
|  | **4** | **A1** | **A1** | **4454** | **6070** | **1616** |
|  | **11** | **A1** | **A1** | **7586** | **8803** | **1217** |
| **NYU119_3** | **1** | **02_AG** | **02_AG** | **903** | **2202** | **1299** |
|  | **2** | **F2** | **F2** | **2203** | **3818** | **1615** |
|  | **4** | **F2** | **F2** | **3996** | **5011** | **1015** |
|  | **5** | **02_AG** | **02_AG** | **5012** | **6633** | **1621** |
|  | **7** | **02_AG** | **02_AG** | **6811** | **8987** | **2176** |
| **MDC179_2** | **1** | **02_AG** | **02_AG** | **837** | **5132** | **4295** |
|  | **2** | **F2** | **F2** | **5133** | **6584** | **1451** |
|  | **4** | **F2** | **F2** | **6761** | **9081** | **2320** |
| **MDC131_3** | **1** | **02_AG** | **02_AG** | **903** | **2092** | **1189** |
|  | **2** | **F2** | **F2** | **2093** | **3834** | **1741** |
|  | **4** | **F2** | **F2** | **3998** | **5024** | **1026** |
|  | **5** | **02_AG** | **02_AG** | **5025** | **6730** | **1705** |
|  | **7** | **02_AG** | **02_AG** | **6840** | **9056** | **2216** |
| **LB104-1** | **6** | **F2** | **F2** | **2591** | **4363** | **1772** |
|  | **10** | **22_01A1** | **22_01A1** | **6223** | **7386** | **1163** |
| **LB089_1** | **2** | **02_AG** | **02_AG** | **1257** | **4935** | **3678** |
|  | **4** | **02_AG** | **02_AG** | **5424** | **6764** | **1340** |
|  | **6** | **02_AG** | **02_AG** | **6895** | **8066** | **1171** |
| **BDHS24_2** | **1** | **F2** | **F2** | **798** | **1728** | **930** |
|  | **3** | **F2** | **F2** | **1786** | **4226** | **2440** |
|  | **6** | **02_AG** | **02_AG** | **4750** | **6610** | **1860** |
|  | **8** | **02_AG** | **02_AG** | **6821** | **9268** | **2447** |
| **NYU6541_6** | **15** | **02_AG** | **02_AG** | **5494** | **6719** | **1225** |
|  | **17** | **02_AG** | **02_AG** | **7166** | **8399** | **1233** |
|  | **18** | **F2** | **F2** | **8400** | **9481** | **1081** |
| **NYU124_2** | **1** | **F2** | **F2** | **889** | **3806** | **2917** |
|  | **3** | **F2** | **F2** | **3992** | **5906** | **1914** |
|  | **6** | **02_AG** | **02_AG** | **6830** | **9236** | **2406** |

**References**

1. Tongo M, Dorfman JR, Martin DP. High Degree of HIV-1 Group M (HIV-1M) Genetic Diversity within Circulating Recombinant Forms: Insight into the Early Events of HIV-1M Evolution. J Virol. 2015;90(5):2221-9.

2. Stamatakis A. RAxML version 8: a tool for phylogenetic analysis and post-analysis of large phylogenies. Bioinformatics. 2014;30(9):1312-3.

3. deCamp A, Hraber P, Bailer RT, Seaman MS, Ochsenbauer C, Kappes J, et al. Global panel of HIV-1 Env reference strains for standardized assessments of vaccine-elicited neutralizing antibodies. J Virol. 2014;88(5):2489-507.

4. Rolland M, Edlefsen PT, Larsen BB, Tovanabutra S, Sanders-Buell E, Hertz T, et al. Increased HIV-1 vaccine efficacy against viruses with genetic signatures in Env V2. Nature. 2012;490(7420):417-20.

5. Courtney CR, Mayr L, Nanfack AJ, Banin AN, Tuen M, Pan R, et al. Contrasting antibody responses to intrasubtype superinfection with CRF02_AG. PLoS One. 2017;12(3):e0173705.
